# Fast-Developing Dynamic Radiative Thermal Management: Full-Scale Fundamentals, Switching Methods, Applications, and Challenges

**DOI:** 10.1007/s40820-025-01676-6

**Published:** 2025-02-17

**Authors:** Long Xie, Xuechuan Wang, Yageng Bai, Xiaoliang Zou, Xinhua Liu

**Affiliations:** 1 College of Chemistry and Chemical Engineering, Institute of Biomass & Functional Materials, Shaanxi University of Science & Technology, Xi’an, 710021 People’s Republic of China; 2 College of Bioresources Chemical and Materials Engineering, Institute of Biomass & Functional Materials, Shaanxi University of Science & Technology, Xi’an, 710021 People’s Republic of China; 3 Key Laboratory of High Performance Plastics, National & Local Joint Engineering Laboratory for Synthesis Technology of High Performance Polymer, Ministry of Education, College of Chemistry, Jilin University, Changchun, 130012 People’s Republic of China

**Keywords:** Thermal comfort, Radiant thermal management, Dynamic radiative thermal management, Renewable energy

## Abstract

Rapid population growth in recent decades has intensified both the global energy crisis and the challenges posed by climate change, including global warming. Currently, the increased frequency of extreme weather events and large fluctuations in ambient temperature disrupt thermal comfort and negatively impact health, driving a growing dependence on cooling and heating energy sources. Consequently, efficient thermal management has become a central focus of energy research. Traditional thermal management systems consume substantial energy, further contributing to greenhouse gas emissions. In contrast, emergent radiant thermal management technologies that rely on renewable energy have been proposed as sustainable alternatives. However, achieving year-round thermal management without additional energy input remains a formidable challenge. Recently, dynamic radiative thermal management technologies have emerged as the most promising solution, offering the potential for energy-efficient adaptation across seasonal variations. This review systematically presents recent advancements in dynamic radiative thermal management, covering fundamental principles, switching mechanisms, primary materials, and application areas. Additionally, the key challenges hindering the broader adoption of dynamic radiative thermal management technologies are discussed. By highlighting their transformative potential, this review provides insights into the design and industrial scalability of these innovations, with the ultimate aim of promoting renewable energy integration in thermal management applications.

## Highlights

- This review comprehensively summarizes the current state-of-the-art in dynamic radiative thermal management technology.
- In-depth discussion of the basic principles of dynamic radiative thermal management technology, design strategies, and a list of related applications are presented.
- An in-depth look at the challenges facing dynamic radiative thermal management technology, providing potential solutions for the future direction of the field.

## Graphical abstract

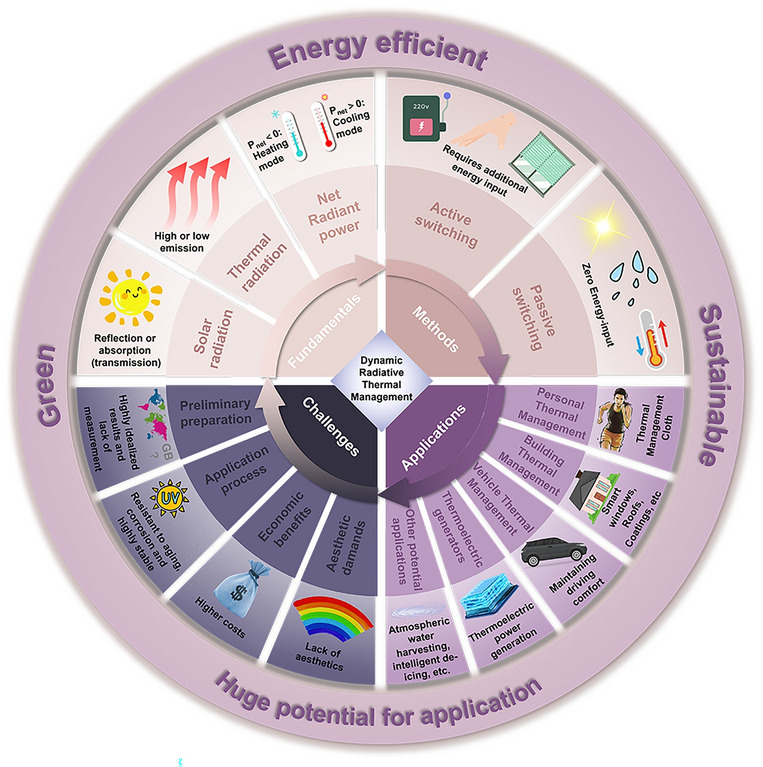

## Introduction

**Fig. 1:**
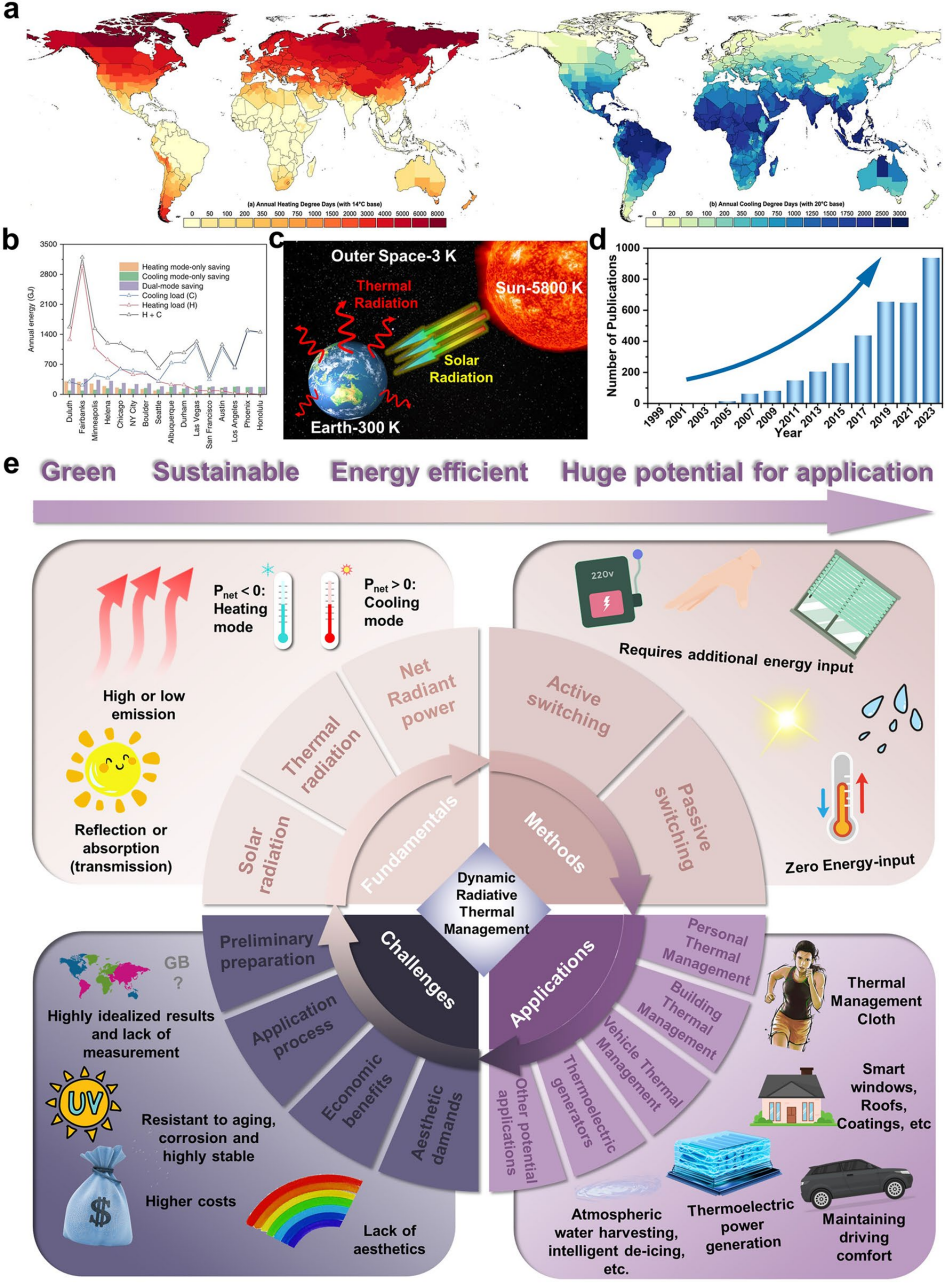
**a** Distribution of global heating and cooling days from 2010 to 2019. [10] Copyright 2020, Springer Nature. **b** Energy consumption and average annual energy savings in US cities under the thermal management model. [18] Copyright 2020, Springer Nature. **c** Schematic of heat transfer between the sun, Earth, and outer space. **d** Statistics of Web of Science publications (search term: “dynamic radiative thermal management”). **e** Overview of dynamic radiative thermal management principles, methods, applications, and challenges

Since the Industrial Revolution, increases in global manufacturing capacity and living standards have driven a rapid rise in energy consumption, compounded by significant population growth. This increased energy demand has resulted in two of the most critical challenges of the twenty-first century: the global energy crisis and climate change [1–5]. Climate change has led to frequent extreme weather events and large fluctuations in ambient temperature, thereby disrupting thermal comfort and causing significant risks to public health [6–8]. To manage the complexity and variability of weather conditions, people have stated to increasingly rely on conventional heating and cooling systems to maintain thermal comfort. However, these systems are typically powered by electricity, leading to substantial energy consumption and significant carbon dioxide emissions [9–14] (Fig. 1a). According to relevant statistics, heating and cooling account for approximately half of global energy consumption, with projected increases of 79% and 83%, respectively, over the next 30 years [15–17]. Furthermore, the economic impact of heating and cooling is expected to reach 2.5% of global GDP by 2030, and climate change is estimated to contribute to approximately 400,000 deaths annually [18, 19] (Fig. 1b). Therefore, there is an urgent need for clean, energy-efficient technologies that can support comfortable and productive living environments, with environmentally friendly radiative thermal management technologies presenting as a promising approach to address these critical challenges [20].

On Earth, objects at temperatures around 300 K can theoretically be heated by the Sun (~ 5800 K) or cooled by exposure to and outer space (~ 3 K) without the need for external energy sources, with the Sun and outer space serving as infinite heat and cold reservoirs, respectively [21–23] (Fig. 1c). In order to fully utilize this advantage, passive radiative cooling (PRC) and solar heating (SH) technologies have been rapidly developed to provide temperature regulation without energy input [24, 25]. PRC harnesses outer space as an efficient heat sink by maximizing sunlight reflection in the 0.3–2.5-μm band and enhancing thermal radiation emission through the 8–13-μm mid-infrared transparent window [26–28]. SH technologies take an opposite approach by maximizing sunlight absorption within the solar spectrum (0.3–2.5 μm) to enhance the material’s heat uptake while also maintaining low emissivity in the mid-infrared range (8–13 μm) to minimize thermal radiation loss and retain absorbed heat efficiently [29, 30]. However, static materials designed for a single thermal management function are unable to adapt to fluctuating weather conditions and may even counteract desired thermal outcomes under certain climates. For instance, radiant cooling materials can inadvertently increase heating demands during cold winters and risk overcooling, while SH materials can drive up cooling requirements in warm environments [31–33]. Therefore, there is an essential need for dynamic, self-adapting radiative thermal management technologies that adjust their function based on environmental changes to enhance energy efficiency and maintain consistent thermal comfort.

Burgeoning dynamic radiative thermal management technologies have demonstrated the ability of these systems to automatically switch between cooling and heating modes in response to ambient temperature changes. This adaptive function requires materials with high solar absorptivity (i.e., high transmittance or low reflectance) and low mid-infrared emittance for heating, while the cooling mode necessitates high solar reflectance (i.e., low transmittance or low absorptivity) and high mid-infrared emittance [34, 35]. Recently, significant efforts have focused on advancing these technologies toward practical applications, and researchers have developed various dynamic radiative thermal management systems utilizing electrochromic [36–38], thermochromic [39–43], mechanically actuated [44, 45], and wetting stimulation [46–48] modes of actuation (Fig. 1d). Although numerous review articles have detailed the principles, materials, and applications of PRC and SH, to our knowledge, comprehensive summaries addressing the development of dynamic radiative thermal management technologies remain limited and lack depth. To support the advancement and optimization of dynamic radiative thermal management materials, we designed this article to provide a timely and detailed review of recent progress in this field.

In this review, we provide a comprehensive summary of recent advancements in dynamic radiative thermal management technology, covering fundamental principles, switching mechanisms, applications, and existing challenges (Fig. 1e). We begin with an overview of the core theories underlying dynamic radiative thermal management. Then, we categorize current dynamic radiative thermal management materials into active and passive types based on their driving mechanisms, offering a detailed analysis within each category. Following this, we review the latest application developments in dynamic radiative thermal management technology, classifying and summarizing them accordingly. Lastly, we discuss the current challenges and future prospects for the field. Collectively, this review provides an extensive and up-to-date reference for researchers and industry professionals, fostering further research and innovation in dynamic radiative thermal management technology while also guiding its practical application in industrial settings.

## Fundamental Principles

### Solar Radiation

The Sun, as a vast source of heat, continuously emits energy. Under clear daytime conditions, solar radiation intensity can reach up to 1000 W m^−2^. However, as solar radiation travels through the atmosphere, its intensity is attenuated due to scattering and absorption by atmospheric particles. For solar radiation within the wavelength range of 0.3–2.5 μm, the absorbed power of a material exposed to solar radiation can be calculated using the following formula:

1 $$ P_{{{\text{solar}}}} = \mathop \smallint \limits_{0.3 \upmu m}^{2.5 \upmu m} \varepsilon \left( {\theta ,\lambda } \right)I_{{{\text{AM1}}{.5}}} \left( \lambda \right){\text{d}}\lambda $$

In the above equation, *θ* represents the angle of incidence of solar radiation, while *ε(θ, λ)* denotes the spectral emissivity of the material at the angle of incidence *θ*. Additionally, *I*_AM1.5_ (*λ*) denotes the specific magnitude of the intensity of the solar spectrum [49]. According to Kirchhoff’s law, when an object is in thermal equilibrium with incident radiation from a blackbody, its absorptivity equals its emissivity, expressed as $\varepsilon \left( {\theta , \, \lambda } \right)\, = \,\alpha \left( {\theta , \, \lambda } \right)\, = \,{1}\, - \,R(\theta , \, \lambda )\, - \,T(\theta , \, \lambda ),$ where *R* and *T* are the reflectivity and transmissivity, respectively. Consequently, the average solar absorptivity and reflectivity can be defined as [50–52]:

2 $$ \overline{\alpha }_{{{\text{solar}}}} = \frac{{\mathop \smallint \nolimits_{{0.3{ }\upmu {\text{m}}}}^{{2.5{ }\upmu {\text{m}}}} \alpha \left( {\theta , \lambda } \right)I_{{{\text{AM}}1.5}} \left( \lambda \right){\text{d}}\lambda }}{{\mathop \smallint \nolimits_{{0.3{ }\upmu {\text{m}}}}^{{2.5{ }\upmu {\text{m}}}} I_{{{\text{AM}}1.5}} \left( \lambda \right){\text{d}}\lambda }} $$

3 $$ \overline{R}_{{{\text{solar}}}} = \frac{{\mathop \smallint \nolimits_{{0.3{ }\upmu {\text{m}}}}^{{2.5{ }\upmu {\text{m}}}} R\left( {\theta ,\lambda } \right)I_{{{\text{AM}}1.5}} \left( \lambda \right){\text{d}}\lambda }}{{\mathop \smallint \nolimits_{{0.3{ }\upmu {\text{m}}}}^{{2.5{ }\upmu {\text{m}}}} I_{{{\text{AM}}1.5}} \left( \lambda \right){\text{d}}\lambda }} $$

For opaque materials, the default *T(θ, λ)* is usually assumed to be 0. Therefore, during PRC, the materials should be designed to reduce sunlight absorption to reduce the heating effect induced by solar radiation but require a sufficiently low solar absorptivity and a sufficiently large *R*_solar_ (infinitely close to 1). In contrast, SH technology requires materials with high solar absorptivity to maximize sunlight absorption for heating purposes, aiming for *α*_solar_ to be as close as 1. Under standard AM 1.5 conditions, where solar intensity reaches up to 1000 W m^−2^, far exceeding the radiant power of a blackbody, modifying thermal radiation within the solar spectrum typically provides sufficient control for thermal management. Many thermochromic and Janus materials use this approach, switching thermal modes by adjusting solar thermal radiation properties. To effectively control *R*_solar_, it is necessary to identify its key influences. Researchers have determined that the primary factor influencing *R*_solar_ is Mie scattering, a phenomenon where incident light is scattered. This effect is especially pronounced when the size of the material closely matches the wavelength of the incident light and when there is a difference between the material’s refractive index and that of its surrounding medium [53]. Sunlight within the visible spectrum (380–780 nm) and near-infrared region (1000–2500 nm) comprises a significant portion of its total intensity. Therefore, introducing suitable micro- and nanostructures can enhance the control of *R*_solar_ based on practical requirements [54]. However, for some transparent materials, *T(θ, λ)* is typically high, allowing most sunlight to pass through. In these cases, reducing light transmission alone can effectively enable switching between thermal management modes, as seen in thermochromic smart windows. The key parameters used to evaluate the performance of smart windows include phase change temperature (*T*_c_), visible light transmittance (*T*_lum_), and solar modulation capability (*ΔT*_sol_). The formulas for calculating these parameters are as follows [55]:

4 $${\tau }_{\text{lum}}=\frac{{\int }_{380\text{ nm}}^{780\text{ nm}}{\varphi }_{\text{lum}}\left(\lambda \right)\tau \left(\lambda \right)\text{d}\lambda }{{\int }_{380\text{ nm}}^{780\text{ nm}}{\varphi }_{\text{lum}}\left(\lambda \right)\text{d}\lambda }$$

5 $${\tau }_{\text{sol}}=\frac{{\int }_{380\text{ nm}}^{2500\text{ nm}}{I}_{\text{AM}1.5}\left(\lambda \right)\tau \left(\lambda \right)\text{d}\lambda }{{\int }_{380\text{ nm}}^{2500\text{ nm}}{I}_{\text{AM}1.5}\left(\lambda \right)\text{d}\lambda }$$

6 $${\Delta \tau }_{\text{solar}}={\tau }_{\text{AM}1.5,\text{ cold}}-{\tau }_{\text{AM}1.5,\text{ hot}}$$

In the above equation, *φ*_lum_*(λ)* represents the standard luminous efficiency function of the human eye, as defined by the International Commission on Illumination, while *τ*_sol_*(λ)* indicates the solar transmittance at varying wavelengths [56]. For windows, normal daylight illumination is essential, and near-infrared light from the sun accounts for about 52% of the total radiant energy [57]. Therefore, researchers have explored alternative strategies and considered tuning materials for solar radiation in the near-infrared band, which represents a potential solution for achieving switchable radiative thermal management techniques.

### Thermal Radiation

Over two thousand years ago, ancient Iranians and Indians developed methods to create and store ice overnight in temperatures above freezing, and in the nineteenth century, researchers observed that certain cotton fabrics cooled to temperatures below ambient levels on clear nights [58, 59]. These examples indicate that, besides solar radiation, another energy transfer mechanism, namely thermal radiation, can also produce a cooling effect under certain conditions. Thermal radiation occurs between all objects with temperatures above absolute zero and spontaneously transfers from warmer to cooler objects, making it a crucial factor in thermal management [60–62]. The thermal radiation power (*P*_rad_) depends on the material’s emissivity and temperature and represents the thermal radiation emitted from an object’s surface. It can be calculated using the following equation:

7 $${P}_{\text{rad}}\left({T}_{\text{c}}\right)=\int \mathit{cos}\theta \text{d}\Omega {\int }_{0}^{\infty }\varepsilon \left(\theta ,\lambda \right){I}_{\text{BB}}\left(\lambda ,{T}_{\text{c}}\right)\text{d}\lambda $$

In this equation, *θ* represents the angle between the incident direction of solar radiation and the vertical direction of the surface, *λ* denotes the wavelength, *ε(θ, λ)* is the emissivity of the material, *T*_c_ indicates the temperature of the material, and *I*_BB_*(λ, T*_c_*)* stands for the intensity of the spectral radiation of the blackbody at the temperature of *T*_c_, which can be defined as:

8 $${I}_{\text{BB}}\left(T,\lambda \right)=\frac{2h{c}^{2}}{{\lambda }^{5}\left({\text{e}}^{hc/\lambda kT}-1\right)}$$

where *h* is Planck’s constant (6.626 × 10^–34^ J s^−1^), *k* is Boltzmann’s constant (1.381 × 10^–23^ J k^−1^), and *c* is the speed at which light propagates in vacuum (2.998 × 10^8^ J s^−1^) [63].

The four basic principles associated with thermal radiation are Kirchhoff’s law, Planck’s law of radiation, Stephen Boltzmann’s law, and Wien’s displacement law. It is well known that when thermal radiation comes into contact with a material, it is usually absorbed, transmitted or reflected, or a combination of these processes, with the total energy transfer equaling the sum of these three components. Stephen Boltzmann’s law can be defined as:

9 $$P=\varepsilon\upsigma {T}^{4}$$

10 $$\alpha \left(\lambda \right)+\tau \left(\lambda \right)+R\left(\lambda \right)=1$$

In this formula, *P* represents the thermal radiation power density, *σ* is the Stephen Boltzmann constant, *T* denotes the surface temperature of the material, and *ε* is the surface emissivity of the material. In addition, the terms *α*, *R,* and *τ* refer to the material’s absorptivity, reflectance, and transmittance, respectively, while *λ* denotes wavelength. The material emissivity *ε(θ, λ)* can be obtained directly from Kirchhoff’s law *α(λ)* = *ε(λ)*.

During thermal management, materials exposed to the external environment are subjected to atmospheric thermal radiation, and the specific radiant power can calculate using the following formula:

11 $${P}_{\text{atm}}\left({T}_{\text{amb}}\right)=\int \mathit{cos}\theta \text{d}\Omega {\int }_{0}^{\infty }\varepsilon \left(\theta ,\lambda \right){\varepsilon }_{\text{atm}}\left(\theta ,\lambda \right){I}_{\text{BB}}\left(\lambda ,{T}_{\text{amb}}\right)\text{d}\lambda $$

where *ε*_atm_ *(θ, λ)* represents the atmospheric directional emissivity at a certain angle and wavelength, which can be obtained from:

12 $${\varepsilon }_{\text{atm}}\left(\theta ,\lambda \right)=1-\tau {\left(\lambda \right)}^{1/\mathit{cos}\theta }$$

In this equation, *τ(λ)* denotes atmospheric transmittance in the direct path. It is worth noting that atmospheric transmittance is influenced by factors such as temperature and humidity, and various atmospheric gas molecules (i.e., N_2_, O_2_, and CO_2_) may have a certain effect on the absorption of thermal radiation. For example, water vapor has absorption bands at 0.94, 1.14, 1.38, and 1.87 μm, CO_2_ has strong absorption bands at 2.7, 4.3, and 15 μm, and certain short-chain alkane gas molecules also exhibit notable infrared absorption [64, 65]. Consequently, the atmosphere shows strong absorption of thermal radiation in specific bands, while thermal radiation outside these absorption bands can be transmitted. The 8–13 μm range, known as the atmospheric transparency window, allows for excellent thermal radiation transmittance [66]. The corresponding emissivity in this window is calculated as follows:

13 $$ \overline{\varepsilon }_{{{\text{LWIR}}}} = \frac{{\mathop \smallint \nolimits_{{8{ }\upmu {\text{m}}}}^{{13{ }\upmu {\text{m}}}} I_{{{\text{BB}}}} \left( {T,\lambda } \right)\varepsilon \left( {T,\lambda } \right){\text{d}}\lambda }}{{\mathop \smallint \nolimits_{{8{ }\upmu {\text{m}}}}^{{13{ }\upmu {\text{m}}}} I_{{{\text{BB}}}} \left( {T,\lambda } \right){\text{d}}\lambda }} $$

For dynamic radiative thermal management, effective PRC requires maximizing the material’s outward thermal radiation, which means the long-wave infrared emissivity (*ε*_LWIR_) should be as close to 1 as possible. In contrast, for SH, *ε*_LWIR_ must be close to 0 to retain the material’s thermal energy by minimizing radiative heat loss. The *ε*_LWIR_ value is primarily determined by the material’s molecular structure. Specific molecular bonds in the mid-infrared range, such as C–O–C (1110–1260 cm^−1^), C–OH (1030–1239 cm^−1^), and C–F_3_ (1148 cm^−1^), exhibit strong vibrational resonances, leading to high infrared absorption and, consequently, a high *ε*_LWIR_ [67, 68].

### Net Radiant Power

As previously discussed, the radiative heat management performance of an object on Earth is primarily influenced by both its outward radiated power and its inward absorbed power. To quantitatively evaluate a material’s radiative heat management performance, an energy balance equation is proposed. According to the law of energy balance, the net heating or cooling power of a material can be calculated using the following equation:

14 $${P}_{\text{net}}={P}_{\text{rad}}-{P}_{\text{solar}}-{P}_{\text{atm}}-{P}_{\text{nonrad}}$$

*P*_net_ represents the net cooling power. A negative *P*_net_ indicates that the radiated power is less than the absorbed power, signifying heating. Conversely, a positive *P*_net_ indicates that the radiated power exceeds the absorbed power, signifying cooling. *P*_nonrad_ denotes the non-radiative heat transfer between the material and its external environment, primarily encompassing thermal convection and conduction. This non-radiative heat transfer can be calculated as follows:

15 $${P}_{\text{nonrad}}=h\left({T}_{\text{amb}}-{T}_{\text{c}}\right)$$

where *h* is the non-radiative heat transfer coefficient, which is largely influenced by environmental conditions [69].

**Fig. 2:**
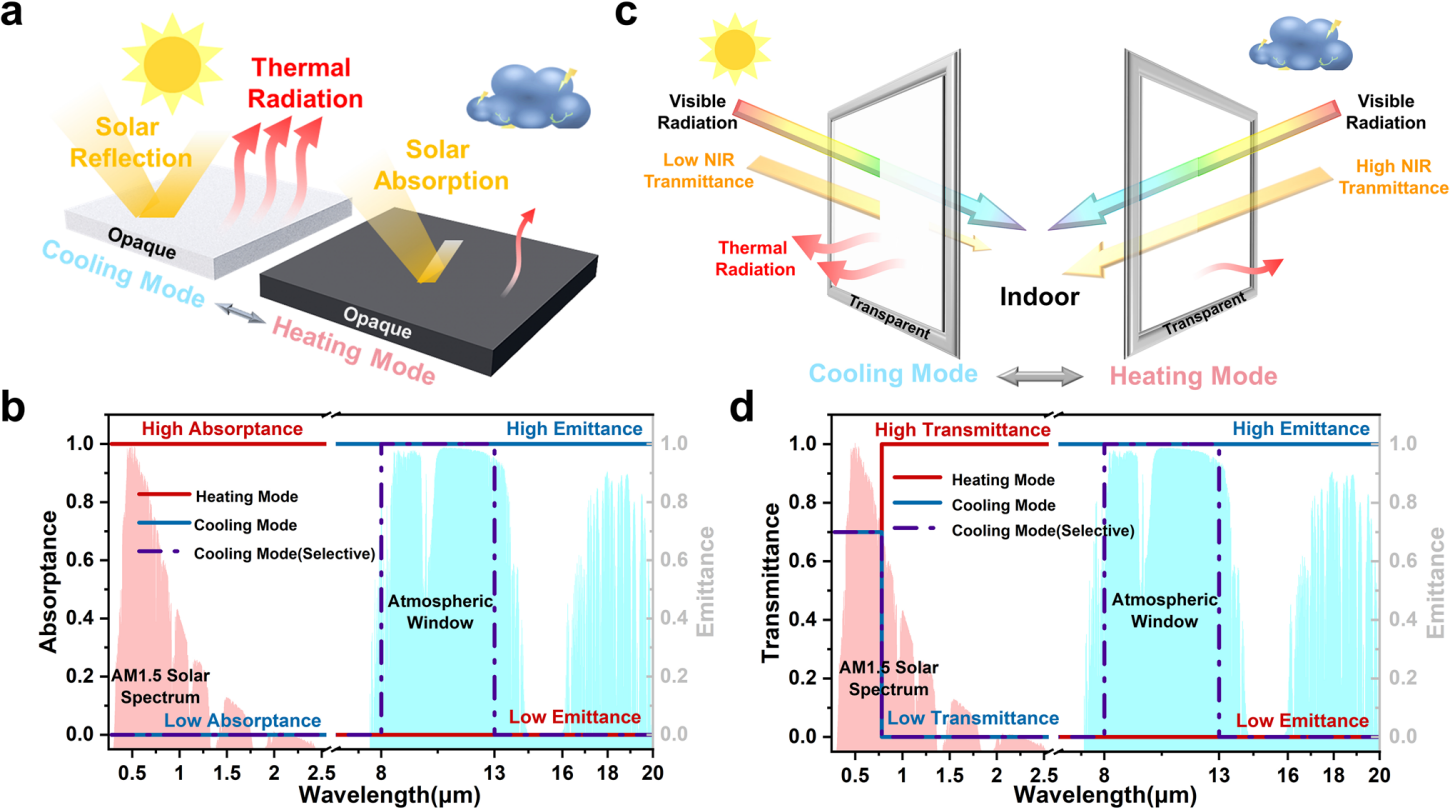
**a** Schematic representation of the radiative thermal management mechanism for opaque material; **b** ideal spectrum for opaque material; **c** schematic representation of the thermal management mechanism for translucent material; **d** ideal spectrum for translucent material

In summary, achieving dynamic radiative thermal management requires two main strategies: modulating the material’s solar radiation absorption or adjusting its thermal radiation properties. For opaque materials, effective cooling during hot seasons necessitates high solar reflectivity and high mid-infrared emissivity to maximize heat dissipation. Conversely, in cold seasons, these materials should reduce mid-infrared emissivity while maximizing solar absorption to retain heat and prevent heat loss (Fig. 2a, b). For transparent smart windows, sunlight transmission should be minimized in hot weather, while near-infrared transmittance should be enhanced in cold weather to increase heat transmission (Fig. 2c, d). However, for smart windows to function effectively in both cooling and heating, visible light transmittance should remain above 60% [57]. It is also worth mentioning that for radiatively cooled materials, they can be categorized into broadband radiative cooling and selective radiative cooling based on their radiative cooling modes in the mid-infrared band [70, 71]. Broadband radiative cooling exhibits high emission performance throughout the mid-infrared band, while selective radiative cooling exhibits high emission only in the atmospheric window (8–13 μm) (Fig. 2b, d). Compared to broadband radiative cooling, selective radiative cooling has superior cooling performance due to the exclusion of downward atmospheric parasitic heat. However, such selective radiative cooling materials are usually very demanding in outdoor environments, and their cooling performance decreases dramatically in real environments, thus limiting their practical applications [72–74].

### Other

In addition to the basic thermodynamic principles mentioned above, entropy balance is also extremely relevant to the design of thermal management systems [75]. This is because heat transfer occurs more or less internally when a thermal management system is cooled or heated. From the second law of thermodynamics, it can be seen that the transfer of heat is often accompanied by entropy changes, so the entropy balance theory is crucial to the thermal management system [76–78]. Entropy balance theory specifically refers to the equilibrium relationship between the entropy input and output in a system and the entropy generated within the system [79]. It is often used to examine the entropy change of a process and thus accurately measure the energy utility of the process, and the commonly used generalized expression is:

16 $${\text{S}}_{\text{In}}-{\text{S}}_{\text{Out}}+{\text{S}}_{\text{Gen}}+{\text{S}}_{\text{Acc}}$$

where S_In_ and S_Out_ represent the two entropy items of the incoming and outgoing systems, respectively, S_Gen_ is the entropy increase due to internal occurrence and consumption (e.g., friction, turbulence, etc.), and S_Acc_ refers to the entropy increase of the system [80].

Only by optimizing the energy conversion process and reducing unnecessary energy loss can the overall efficiency of thermal management be improved. Therefore, in the actual design of thermal management system, the entropy balance state of the system should be fully considered to ensure that the temperature requirements are met while realizing efficient and energy-saving operation.

## Switching Method

**Fig. 3:**
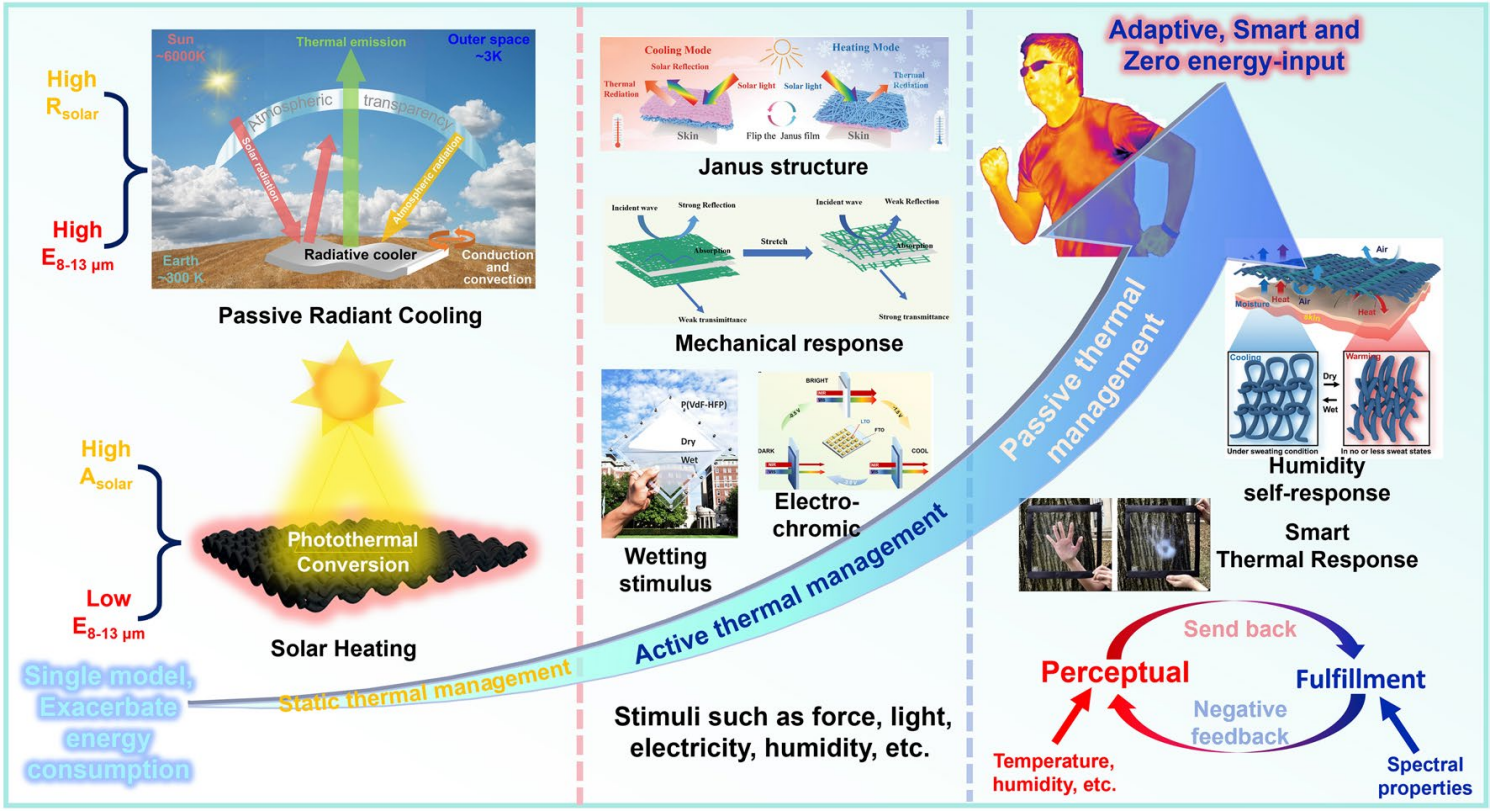
Summary of developments in thermal management technology

Dynamic radiative thermal management technology can achieve adjustable heating and cooling effects by enabling materials to switch their thermal management mode in response to ambient temperature changes, thereby expanding their potential applications. In this regard, researchers have developed various materials and devices with dynamic radiative thermal management capabilities, which can be broadly categorized as either active or passive based on their switching mechanisms (Fig. 3). Active switching is not spontaneous and requires human intervention, such as manual flip, mechanical pressure, and voltage stimulation [81–84]. In contrast, the passive type does not require human intervention, and the material or device can spontaneously switch thermal management modes in response to environmental stimuli such as light, temperature, and humidity [85–90].

### Active Switching

#### Physical Devices

To enable controllable switching of radiative thermal management modes, researchers have developed simpler and more environmentally friendly switchable radiative thermal management devices based on the knowledge of relevant optical theories and the use of common commercially available materials. The specialized structural design of these devices allows flexible switching between thermal management modes when activated by human intervention. Although these devices require external triggering, they are not limited to a specific temperature range, as is the case with thermochromic materials. Instead, their thermal management mode can be adjusted through intentional human input, making large-scale production applications feasible. Based on structural design and operational mechanisms, these devices can be classified into three main types: shutter structures [91], contrast refraction structures [92], and mechanical reconfiguration structures [93].

**Fig. 4:**
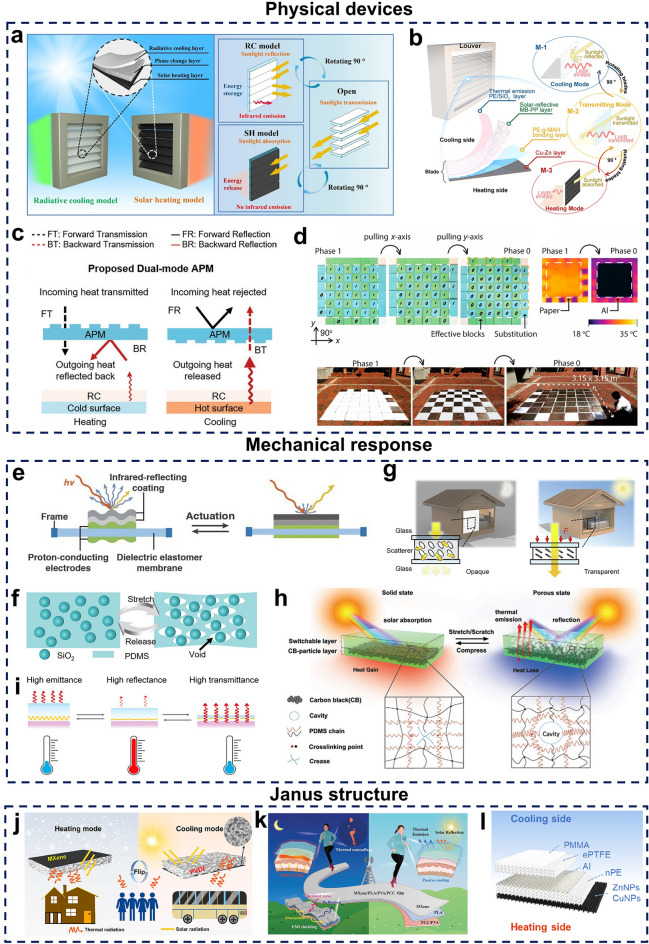
**a-d** Schematic representations of device structures for dynamic radiative thermal management. **a** Shutter structure. [96] Copyright 2023, American Chemical Society. **b** Shutter with energy storage properties. [97] Copyright 2022, American Chemical Society. **c** Action principle of dual-mode asymmetric photon reflector. [99] Copyright 2022, Wiley–VCH. **d** Mechanically reconfigured structure. [93] Copyright 2023, American Chemical Society. **e-i** Mechanically responsive design strategies for dynamic radiative thermal management. **e** Modulation principles based on octopus and jellyfish. [102] Copyright 2018, AAAS. **f** Pleated structures. [103] Copyright 2024, Wiley–VCH. **g** Thickness-activated pleats. [104] Copyright 2024, Springer Nature. **h** Nanomaterial composites. [105] Copyright 2020, Wiley–VCH. **i** Metal-based composites. [106] Copyright 2021, American Chemical Society. **j-l** Janus structures. **j** Bilayer structures. [107] Copyright 2023, American Chemical Society. **k** Sandwich structures. [108] Copyright 2023, Wiley–VCH. l) Multilayer structures. [109] Copyright 2021, American Chemical Society

Shutter structures are usually made of several strips of material arranged in parallel, where light intensity can be adjusted by changing the angle of the blades [94, 95]. Based on this design, some researchers have chosen to integrate radiatively cooled and solar-heated materials into the blades, allowing radiative thermal management mode switching simply by rotating the blades with an actuator [96] (Fig. 4a). Considering the properties of phase change materials for storing and releasing latent heat, some scholars have also introduced phase change materials into switchable radiative thermal management blades to create multifunctional devices that combine heating, cooling, and thermal energy storage [97] (Fig. 4b). In addition, asymmetric infrared transmission and reflection are also recognized as important advancements for achieving all-season and all-terrain sustainable passive cooling/heating technologies [98]. Contrast refractive structures, as the name suggests, utilize the difference in refractive indices of different media to enable the modulation of radiative thermal management modes. For example, a dual-mode asymmetric photon reflector (APM) made of a silicon-based diffraction grating demonstrates asymmetric broadband infrared transmission and reflection at forward and reverse incidences, which creates an asymmetric radiative heat transfer channel, facilitating outward heat transfer for cooling and inward heat transfer for heating [99] (Fig. 4c). Finally, mechanically reconfigurable structures leverage innovative designs such as 2D-3D spatial rearrangement to achieve unique physical properties [100, 101]. This approach inspired the development of a switchable surface consisting of vertically interwoven bands on the x–y plane. By dynamically adjusting the overlap sequence of these bands, selective spectral modulation is achieved, allowing control over thermal management modes [93] (Fig. 4d).

#### Mechanical Response

Mechanically responsive materials can change their optical properties in response to simple mechanical strains, such as compression or tension. This behavior arises because their surface morphology or internal structure is reconfigured or deformed when mechanical actuation occurs, thus changing its scattering efficiency to enable optical modulation [110]. Polymer materials, due to their strong viscoelastic interactions between internal molecular chains, are well suited for mechanical responsiveness. However, the inherent performance of polymer materials is often insufficient for use in switchable radiative thermal management, limiting their practical applications. To address this limitation, researchers have attempted several enhancements, such as integrating micro- and nanostructures or incorporating specialized functional materials. Based on design strategies, mechanically responsive materials can be categorized into pleated structures, nanomaterial-based composites, and metal-based composites [111–114].

In nature, certain marine organisms, such as jellyfish and octopuses, can adjust their transparency and color by modulating the elongation or contraction of tissues and pigment cells, allowing them to blend seamlessly with their surroundings [102] (Fig. 4e). Based on such capability, researchers have designed pleated structures that modulate incident light by laminating a flexible substrate with a relatively rigid thin film, creating folds during mechanical deformation [103] (Fig. 4f). However, mechanically responsive materials based on pleated structures often require a large operating space, posing limitations for practical applications. Switching the activation mode from traditional in-plane deformation to a through-thickness compression helps address this challenge by reducing space requirements [104] (Fig. 4g). Although pleated structures are applicable to most elastomers, their deformation direction is extremely limited and optical modulation through light scattering occurs only at the elastomer’s surface, resulting in a narrow range of optical modulation. To address this issue, researchers have leveraged the interactions between light and the internal microstructure of elastomers to modulate optical transmittance. During the cross-linking process, silicon coatings form substable folds. When simple mechanical stimuli, such as stretching, scraping, or pressing, are applied, cavities are created within these substable folds, enabling reversible modulation between porous and transparent solid states [105] (Fig. 4h). Additionally, metallic reflectors have been integrated with elastomers, taking advantage of the high reflectivity and low emissivity properties of metals in the mid-infrared spectrum. By altering the metal coverage on the substrate through simple stretching and releasing actions, the device can switch between emission, reflection, and transmission modes [106] (Fig. 4i).

However, it is important to note that the large-scale implementation of mechanically responsive switchable radiative thermal management modes continues to face substantial challenges. First, controlling the degree of stretching or compression with precision remains difficult, which can significantly impact practical applications. Second, it is uncertain whether repeated mechanical responses might degrade the material’s dynamic radiative thermal management performance over time. Finally, large-scale stretching or compression is inherently challenging to control and introduces additional energy consumption. Furthermore, the influence of external environmental factors, such as weather conditions, humidity, and wind speed, on the actual thermal management performance requires further investigation.

#### Janus Structure

Integrating both cooling and heating functionalities within a single material presents significant challenges; however, the advent of Janus structures has provided a promising solution. The term "Janus" is inspired by the Roman god with two faces, symbolizing the ability to look into both the past and the future [115, 116]. Drawing from this concept, researchers have developed various Janus materials with distinct properties, such as Janus wettability and charge differentiation, and Janus thermal management materials being among the most representative ones [117–120]. Based on their unique layered configurations, Janus materials can be classified into bilayer, sandwich, and multilayer structures.

Bilayer designs are especially prevalent among Janus materials with all-seasonal thermal management properties, as they are simpler to fabricate than sandwich and multilayer structures. In addition, this structural simplicity not only reduces preparation and production costs but also appeals to researchers due to the relative ease of manufacturing [121–123]. For example, coral-like porous polyvinylidene fluoride (PVDF) films loaded with MXene on one side have been shown to exhibit all-weather thermal management properties [107]. In these composite films, the porous structure enhances light reflection and scattering on the cooling side, while MXene addition improves light absorption on the heating side and provides Joule heating properties (Fig. 4j). However, it should be noted that despite their cost-effectiveness and simplicity, bilayer Janus structures often face challenges related to weak interlayer adhesion and limited mechanical strength [124]. To overcome these limitations, researchers have explored the use of a base layer with high adhesion or mechanical strength to create radiative thermal management materials with sandwich structured [125, 126]. For instance, a multifunctional wearable Janus film with all-season thermal management was created by combining continuous electrostatic spinning with spraying, using poly(lactic acid) (PLA) as a substrate with modifications on both sides (Fig. 4k). The radiatively cooled layer’s unique sugarloaf structure significantly enhances the cooling effect, while the PLA substrate improves the film’s mechanical strength [108]. Apart from the bilayer and sandwich structures, multilayer configurations are also common, but although these structures require more complex fabrication processes, the inclusion of multiple functional layers provides additional properties, such as breathability, water resistance, and flexibility, which expand the range of potential applications [109] (Fig. 4l).

#### Electrochromic

**Fig. 5:**
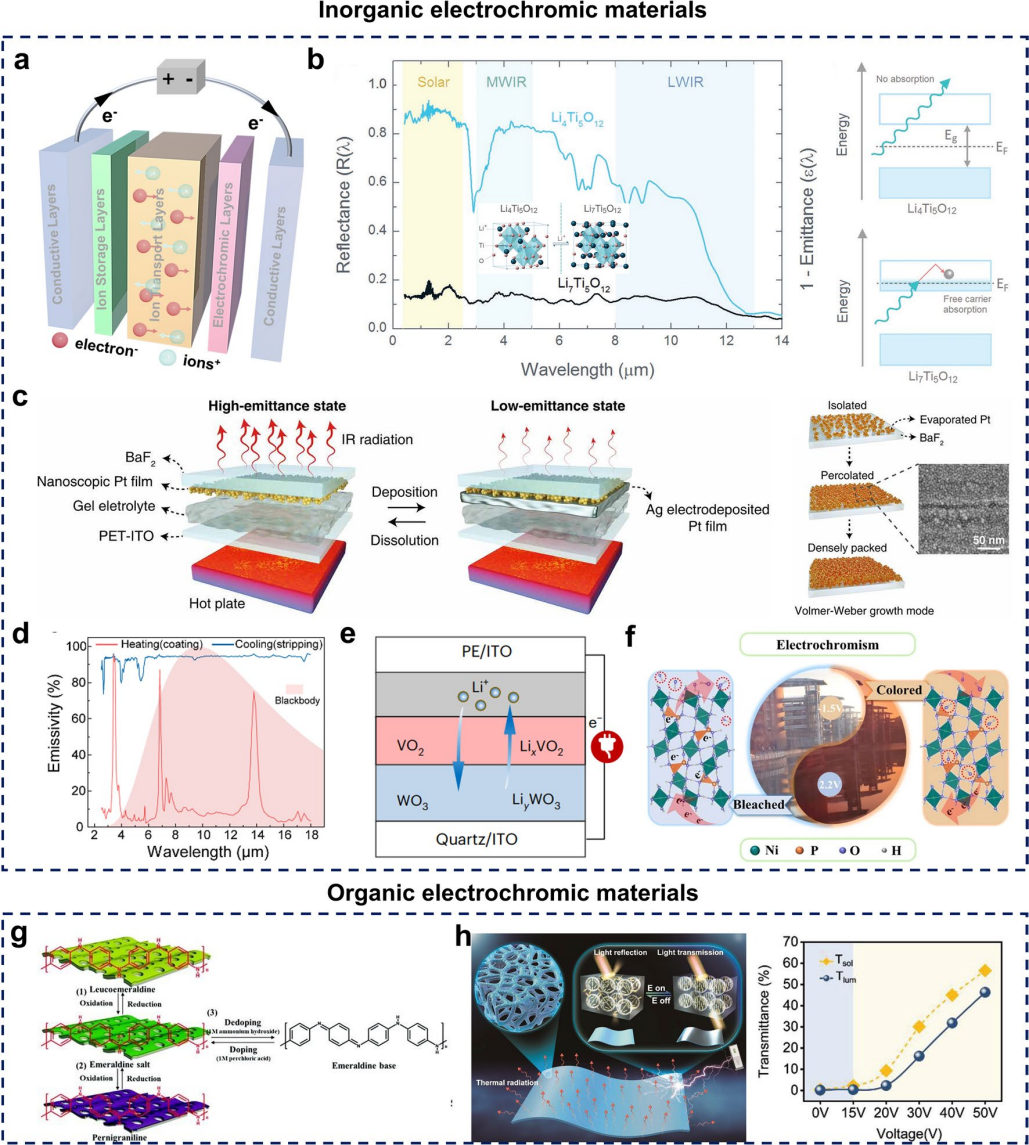
**a** Basic component of electrochromic devices; **b-f** devices utilizing inorganic electrochromic materials. **b** LTO electrochromic structures with spectral properties. [133] Copyright 2018, Wiley–VCH. **c** Metal-based electrochromic devices. [134] Copyright 2020, AAAS. **d** Electrochromic devices with combined use of multi-metals. [135] Copyright 2021, American Chemical Society. **e** Electrochromic devices based on excessively metal oxides. [136] Copyright 2024, Springer Nature. **f** Electrochromic devices based on inorganic compounds. [137] Copyright 2023, Springer Nature. **g-h** Devices utilizing organic electrochromic materials. **g** Mechanism of PANI-based electrochromic devices. [138] Copyright 2019, Royal Society of Chemistry. **h** PDLC-based electrochromic devices. [139] Copyright 2023, Wiley–VCH

Electrochromism is characterized by the reversible alteration of a material’s optical properties in response to an applied electric field [127]. Due to their unique optoelectronic modulation capabilities, low-voltage operation, and remarkable durability, electrochromic materials have become integral to dynamic radiative thermal management applications, including energy-saving windows, smart buildings, and more [128–130]. In practical applications, electrochromic materials are typically incorporated into electrochromic devices (ECDs), which can function in either transmissive or reflective modes. A typical ECD comprises four key components: a transparent conductive layer, an ion-transport layer, an ion-storage layer, and an electrochromic layer [131] (Fig. 5a). There is a wide variety of electrochromic materials, and based on their composition, they can be classified into inorganic electrochromic materials and organic [132].

Inorganic electrochromic materials primarily modulate spectral properties through electrochemical ion insertion and extraction. Common examples include lithium titanate (LTO), metals, transition metal oxides, and various inorganic compounds [140–143]. LTO, known for its high reversibility in Li^+^ insertion, undergoes a transformation from a broadband semiconductor to a metallic state after Li^+^ is inserted. In this state, the nanoscale LTO on metallic substrates shifts from an optically reflective to a solar-absorbing and thermally emissive state (Fig. 5b), achieving modulation capabilities across the solar, mid-infrared (MWIR), and long-wavelength infrared (LWIR) spectra, with values of 0.74, 0.68, and 0.30, respectively [133]. Metallic materials, such as Ag and Cu, possess high reflectivity and can achieve optical property modulation via reversible electrodeposition, which facilitates the deposition or dissolution of metal particles on the electrode surface. For example, Ag deposition on platinum (Pt) nanofilms, known for their high infrared (IR) absorption and partial IR transmission, enables the device to alternate between states of high and low reflectivity [134] (Fig. 5c). To further enhance spectral modulation, combining multiple metals has proved to be an effective strategy [135] (Fig. 5d). Among transition metal oxides, tungsten trioxide (WO_3_), vanadium dioxide (VO_2_), and manganese dioxide (MnO_2_) are prominent, with WO_3_ being particularly notable due to its multiple oxidation states, non-toxic and environmentally benign nature, high optical modulation efficiency, and cost-effectiveness [144]. Modulation of the embedding depth of lithium ions in vanadium oxide/tungsten oxide (VO_2_/WO_3_) structures can transform monoclinic VO_2_/WO_3_ into rutile Li_x_VO_2_ and cubic Li_y_WO_3_ when a voltage is applied to achieve modulation of near-infrared and visible light [136] (Fig. 5e). Despite the advantages of metallic oxides, their limited electronic conductivity and insufficient active sites continue to restrict their broader application in electrochromism [145]. Prussian blue (PB) and transition metal phosphates, such as NiHPO_4_·3H_2_O, are highly electrochemically active inorganic compounds and are therefore considered promising candidates for advanced electrochromic materials [137, 146] (Fig. 5f).

Inorganic electrochromic materials are costly and brittle, limiting their utility in large-scale production and application. Consequently, organic electrochromic materials, which are lightweight, flexible, easy to process, and possess high contrast, fast response times, and dynamic color-changing capabilities, have garnered considerable interest [147–150]. Organic electrochromic materials predominantly rely on conductive polymers and polymer-dispersed liquid crystals. Conductive polymers, in particular, facilitate reversible light modulation through polarization exciton generation and redox reactions, while polymer-dispersed liquid crystals (PDLCs) leverage voltage-driven refractive index differences between liquid crystals and polymers [151–153]. Among conductive polymers, polyaniline (PANI) and poly(3, 4-ethylenedioxythiophene) (PEDOT) derivatives are the most common, with PANI being widely studied [154, 155]. When a voltage within the range of − 0.2 to 0 V is applied, PANI remains in a reduced state and displays a transparent yellow-white-rose coloration (LB). At approximately 0.4 V, LB undergoes partial oxidation, transforming into a green emeraldine salt (ES) form (Fig. 5g). Further oxidation of ES within a voltage range of 0.6 to 0.9 V induces a blue hue, eventually forming a violet perovskite structure (PB) [138]. Polymer-dispersed liquid crystals (PDLCs), a novel class of electrochromic materials, have increasingly captured researchers’ interest in recent years. The arrangement of liquid crystals within PDLCs is influenced by an applied electric field, enabling reversible switching between transparent and opaque states. However, reports on achieving precise, on-demand control of the thermal management efficiency in PDLCs remain limited [156, 157]. In a pioneering study, researchers introduced a mid-infrared-emitting monomer into a PDLC matrix, demonstrating that on-demand, multistage tuning of electro-optical performance and thermal management efficiency could be achieved, which was accomplished by adjusting the concentration of mid-infrared-emitting components, film thickness, and microscopic morphology [139] (Fig. 5h).

Despite the numerous advantages of electrochromic materials, their limitations, such as poor cyclic durability, high cost, and limited flexibility, pose substantial challenges [158]. Furthermore, large-scale application of electrochromics remains difficult due to the absence of efficient, scalable production methods, and the energy demands associated with voltage stimulation are considerable. Although some researchers have developed ECDs with integrated energy storage capabilities, the results have yet to demonstrate a significant impact.

#### Chemical Stimulus

**Fig. 6:**
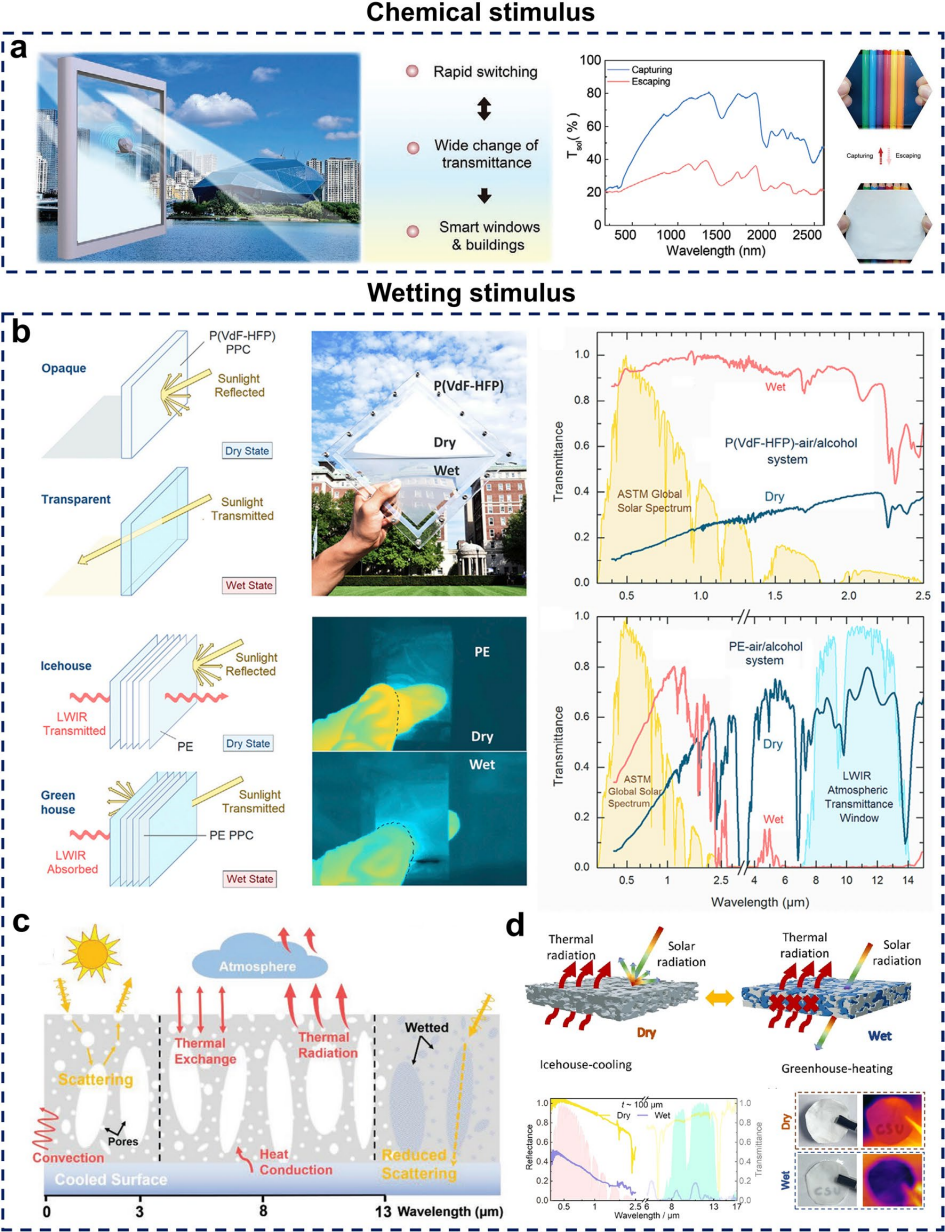
**a** Response of materials to chemical stimuli. [160] Copyright 2023, Wiley–VCH. **b-d** Responses to wetting stimulus. **b** Wetting stimulus response in porous polymer-based materials. [161] Copyright 2019, Elsevier. **c** Schematic diagram of the wetting stimulus response with vertically aligned microporous structure to overcome thickness limitations. [162] Copyright 2022, Wiley–VCH. **d** Wetting stimulus-responsive material with capabilities for full-spectrum modulation. [163] Copyright 2024, American Chemical Society

Conventional materials used for all-season thermal management are often hindered by slow switching speeds, limited cycling durability, and insufficient mechanical strength, which greatly restrict their practical applications. Recent studies indicate that certain materials when exposed to chemical stimuli such as organic solvents undergo internal or structural transformations that consequently alter their optical properties [159]. These materials typically demonstrate rapid response rates and high mechanical strength, making them promising candidates for thermal management applications, including smart windows. One innovative approach involves designing smart alcohol-responsive gels through supramolecular configurations (Fig. 6a). When exposed to ethanol molecules, the supramolecular structure of this gel undergoes conformational changes, allowing it to achieve effective light transmittance modulation with fast switching times [160]. However, this method currently faces two primary limitations: firstly, the spectral modulation range remains relatively narrow, with limited research available on its impact on emissivity spectra; secondly, the solvent consumption is high, and recovery is not feasible, leading to increased operational costs.

#### Wetting Stimulus

In addition to the active switching methods previously discussed, solvent-induced reversible wetting offers an additional strategy for switching thermal management modes [164–166]. This approach leverages the properties of polymers with reversible wetting capabilities, which often possess a porous structure with pore sizes ranging from 0.1 to 10 μm. The highly porous nature of these materials results in substantial Mie scattering across various wavelengths, contributing to high solar reflectivity and a distinct bright white appearance [167]. However, when a liquid with a refractive index similar to that of the polymer infiltrates the material, the refractive index contrast decreases, reducing Mie scattering efficiency. Therefore, the material becomes transparent, permitting incident light to pass through [168]. This refractive index matching allows radiative cooling materials with porous structures to effectively control thermal management modes. In a pioneering study, Mandal et al. employed this refractive index matching principle to dynamically regulate the solar transmittance and thermal radiation of porous polymer materials, and it is also noteworthy that a diverse range of polymer materials can exhibit this reversible wetting property [161] (Fig. 6b). For reversibly wetted porous polymer materials, specific thickness constraints are required to achieve both high solar reflectance and high transmittance. However, the optimal thicknesses for these two properties are inherently contradictory, presenting a significant challenge for researchers, and this limitation can be addressed by creating a hierarchical porous coating composed of vertically aligned micropores within a nanoscale porous matrix through a solvent exchange process, which effectively broadens the material’s applicability [162] (Fig. 6c). Although this approach overcomes thickness limitations, the thermal modulation range of reversibly wettable materials remains concentrated in the solar wavelength band, restricting their capacity for full-spectrum modulation from solar to thermal radiation [169, 170]. The use of structured polyethylene for dynamic radiative thermal management enables simultaneous modulation of both solar and thermal radiation, achieving optimal spectral performance at a film thickness of just 100 μm [163] (Fig. 6d).

### Passive Switching

#### Thermochromic

Thermochromic materials, which undergo phase transition or discoloration with temperature changes, and whose optical properties change due to changes in crystal or molecular structure, make them promising candidates for dynamic radiative thermal management applications [171–173]. In contrast with active switching materials, thermochromic materials do not require external energy input to modulate thermal management modes, aligning well with low-carbon and environmentally sustainable principles. This energy-efficient switching ability has garnered significant interest from researchers. Presently, seven primary types of thermochromic materials have been extensively studied, namely vanadium dioxide (VO_2_) [174–176], germanium-antimony telluride (GST) [177], chalcogenides [178, 179], hydrogels [180–184], ionic gels [185], thermochromic microcapsules [186–188], and salted polymers [189].

##### ***VO***_***2***_

**Fig. 7:**
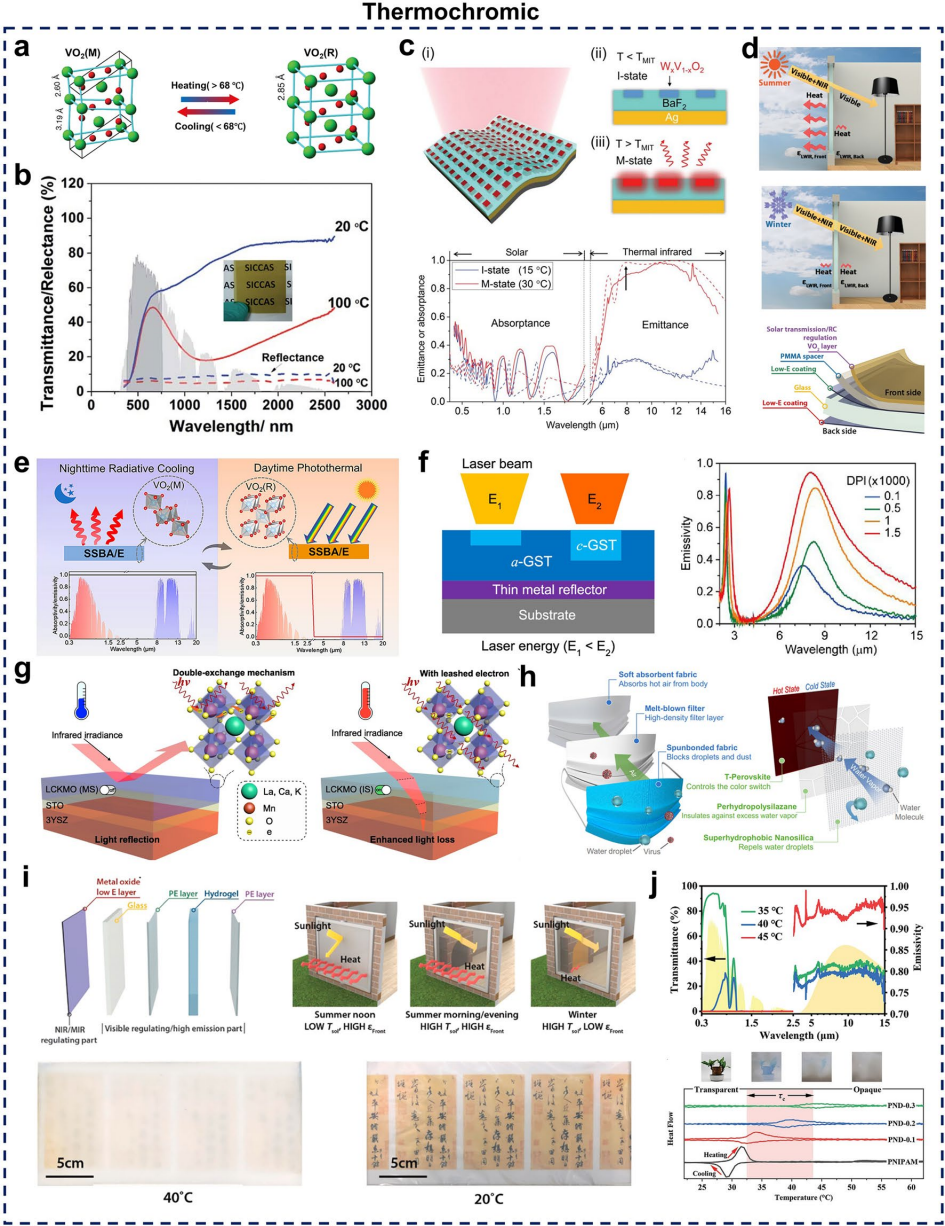
**a-e** VO_2_-based thermochromic materials. **a** Schematic representation of VO_2_ structure before and after phase change transition. [191] Copyright 2021, Elsevier. **b** Spectral properties of VO_2_-based phase change materials at 20 and 100 °C. [194] Copyright 2024, Springer Nature. **c** VO_2_-based Fabry–Perot (F-P) resonance structure. [197] Copyright 2021, AAAS. **d** VO_2_-based F-P resonance structure with polymer as a dielectric layer. [198] Copyright 2021, AAAS. **e** VO_2_-based thermal management device with energy harvesting functionality. [199] Copyright 2023, American Chemical Society. **f** GST-based thermochromic planar cavity structure. [200] Copyright 2024, American Chemical Society. **g, h** Thermochromic chalcogenide materials. **g** Schematic of thermochromic chalcogenide structure. [201] Copyright 2022, Elsevier. **h** Mask-inspired thermochromic chalcogenide window with a unique triple-layer configuration. [202] Copyright 2024, Springer Nature. **i, j** Thermochromic hydrogel materials. **i** Thermochromic hydrogel smart window with tunable solar transmittance and thermal emissivity. [203] Copyright 2021, Elsevier. **j** Thermochromic hydrogel smart window with an extended transition temperature range. [204] Copyright 2023, Wiley–VCH

VO_2_ is a widely studied inorganic thermochromic material due to its phase transition temperature (68 °C), which is closer to ambient conditions than other inorganic materials [190]. Below this phase transition temperature, VO_2_ exists in a monoclinic (M) insulating phase and is characterized by low long-wavelength infrared (LWIR) absorption. When the temperature exceeds the phase transition threshold, VO_2_ transforms into a rutile (R) metallic phase, which exhibits high LWIR absorption and emissivity while maintaining nearly unchanged optical properties in the visible spectrum [191] (Fig. 7a). Utilizing this property, researchers have lowered the phase transition temperature by doping pure VO_2_ with materials such as molybdenum (Mo) or tungsten (W) [192, 193] and combining it with solar reflective materials, which allows for the modulation of the optical properties within the NIR spectrum, which has shown promising applications in dynamic radiative thermal management [194] (Fig. 7b). VO_2_-based thermochromic materials are often designed with Fabry–Perot (F-P) resonance structures, which provide spectral tunability by incorporating an infrared transparent dielectric layer between the VO_2_ and metallic layers [195, 196]. In a notable advancement, two-dimensional photolithographic W_x_V_1-x_O_2_ arrays were embedded within a BaF_2_ dielectric layer atop an Ag film. This configuration, combining temperature-driven metal–insulator transition with photon resonance properties, achieved spontaneous emissivity switching from 0.20 to 0.90 [197] (Fig. 7c). Polymers can also serve as dielectric layers, for instance, using polymethylmethacrylate (PMMA) as a dielectric layer maintains high transparency for smart window applications, with an ε_LWIR_ modulation capability of 0.4 [198] (Fig. 7d). To address the limitations of conventional VO_2_-based materials, which do not efficiently harness or utilize energy, researchers have developed a spectrally selective broadband absorber/emitter (SSBA/E) (Fig. 7e). This innovative design captures heat from photothermal conversion during the day and repurposes it for nighttime cooling, thus enhancing overall cooling efficiency [199].

##### GST-Based Thermochromic Materials

GST is a phase change material that can reversibly transition between amorphous (a-GST) and crystalline (c-GST) states when exposed to conditions such as heating or applied voltage. At low temperatures, the amorphous structure (a-GST) exhibits high phototransparency, whereas the crystalline structure at elevated temperatures (c-GST) demonstrates strong absorption in the MIR region [205, 206]. Unlike the highly oxidizable VO_2_, GST films are highly stable, making them a promising thermochromic material for dynamic thermal management applications [207]. A planar cavity structure utilizing GST thin films with adjustable emissivity has been developed, where ε_LWIR_ can be controlled from 0.15 to 0.77 by adjusting the laser energy applied to the GST film via a UV laser beam [200] (Fig. 7f).

##### Chalcogenide

Thermochromic chalcogenide materials have gained significant attention in recent years. Similar to GST, their low formation energy enables rapid and precise responses to temperature fluctuations [201] (Fig. 7g). These materials transition from a high-transmittance heating state at lower temperatures to a low-transmittance cooling state at higher temperatures, providing considerable potential for energy-saving applications [208–210]. However, the environmental stability of chalcogenides is limited, and they are prone to humidity-induced degradation, which restricts their viability for use as smart windows. To address this limitation, a unique three-layer thermochromic chalcogenide window inspired by medical mask design was developed (Fig. 7h). This window triggers thermochromic color change through water vapor transmission while effectively repelling moisture, thus significantly extending its durability [202].

##### Hydrogels

Thermochromic hydrogels are increasingly used in thermochromic smart windows due to their advantages of low cost, high transparency, broad spectral modulation range, and ease of preparation [211–216]. These hydrogels function based on the principle that thermosensitive polymers exhibit different hydrophilic properties above and below the low critical solution temperature (LCST). Specifically, when the external temperature is below the LCST, numerous hydrogen bonds form between polymer chains and water molecules, resulting in a uniform dispersion of polymer chains and high transparency. Conversely, when the temperature rises above the LCST, hydrogen bonds break, causing the polymer chains to aggregate and transition to an opaque state [217, 218]. Common thermochromic hydrogels include poly(N-isopropylacrylamide) (PNIPAM) [219–221], poly(N-vinylcaprolactam) (PNVCL) [222], hydroxypropylmethylcellulose (HPMC) [223], hydroxypropylcellulose (HPC) [224], methylcellulose (MA) [225], poly(hydroxy) propyl acrylate (PHPA) [226] and polyvinyl butyral (PVB) [227], and others.

Traditional thermochromic hydrogel smart windows can modulate the solar spectrum but lack emissivity modulation capabilities. To address this limitation, Wang et al. developed a novel thermochromic smart window capable of tuning both solar transmission and thermal emissivity for the first time [203] (Fig. 7i). This window not only demonstrated excellent solar modulation but also achieved an emissivity tunability of 0.85. Furthermore, to overcome limitations associated with narrow operational temperature ranges and moderate energy efficiency that restrict applicability across diverse climates, researchers adjusted the hydrophilicity and microstructure of hydrogels to enhance solar modulation. This innovation led to the development of smart windows with a broad temperature range (32.5–43.5 °C), enabling effective building thermoregulation across different climates and under various weather conditions [204] (Fig. 7j).

##### Ionic Gels

**Fig. 8:**
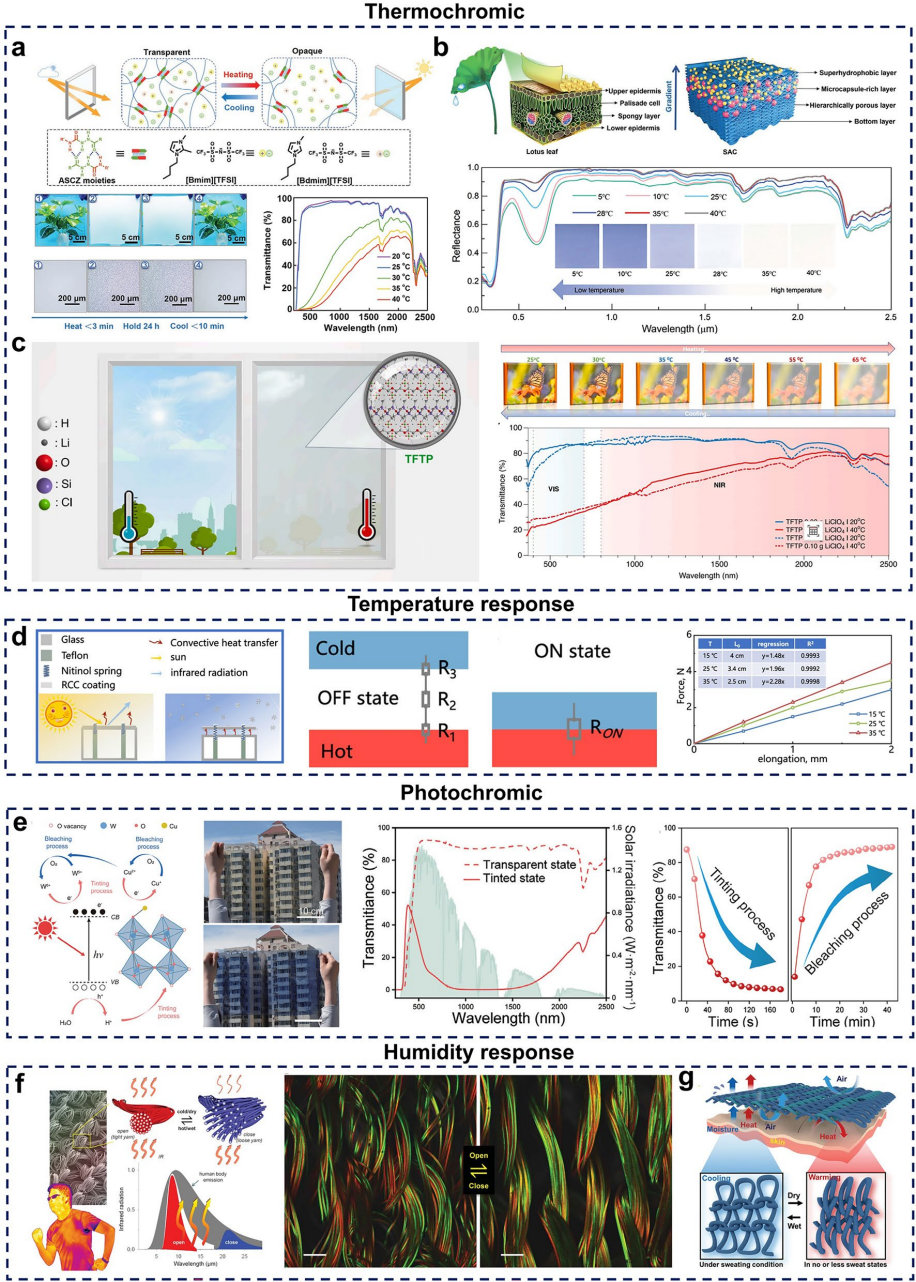
**a-c** Thermochromic materials. **a** Thermochromic ionic gel. [232] Copyright 2023, Wiley–VCH. **b** Thermochromic microcapsules. [233] Copyright 2024, Wiley–VCH. **c** Thermochromic salted polymers. [189] Copyright 2024, Elsevier. **d** Temperature-responsive devices. [234] Copyright 2022, American Chemical Society. **e** Photochromic materials. [235] Copyright 2023, Wiley–VCH. **f**, **g** Humidity-responsive materials. **f** Humidity-triggered bimorphic fibers. [236] Copyright 2019, AAAS. **g** Humidity-responsive shutter-like fabrics with dynamic thermal management capabilities. [237] Copyright 2024, Wiley–VCH

Although thermochromic hydrogels offer several advantages, their environmental stability can be compromised by issues of water evaporation and freezing [228]. In contrast, ionic liquid-based gels exhibit superior environmental stability due to their low volatility and wide temperature tolerance [229]. Certain ionic liquid gels have been shown to exhibit thermochromic properties when exposed to external stimuli, such as light and heat, which makes them promising candidates for thermochromic smart window applications [230, 231]. For instance, by incorporating binary ionic liquids (ILs) into a specially designed self-healing polyurea matrix with reversible, multiple hydrogen-bonded acylamino nitrogen (ASCZ) groups, researchers created a self-adhesive and self-healing thermochromic ionic gel, which demonstrated excellent environmental stability, significantly enhancing its feasibility for long-term smart window applications [232] (Fig. 8a).

##### Thermochromic Microcapsules

In nature, there are many animals, such as chameleons and rain frogs, which can spontaneously change colors according to their environment to avoid predators or maintain their body temperature [238–240]. Inspired by these biological mechanisms, researchers have developed thermochromic microcapsules capable of dynamic color changes and function based on internal electron transfer induced by temperature changes, which leads to structural adjustments and visible color shifts [241]. This thermochromic property allows microcapsules to modulate reflectivity according to ambient temperature, presenting great potential for dynamic radiative thermal management applications. Accordingly, thermochromic microcapsules have been integrated into thermal management materials, leading to the creation of various adaptive thermal management solutions [242, 243]. One example includes flexible, adaptive coatings produced by embedding thermochromic microcapsules during the phase separation process (Fig. 8b). It is worth mentioning that these coatings are superhydrophobic, significantly enhancing durability and making them particularly suitable for prolonged outdoor applications [233].

Despite the progress in developing thermochromic microcapsule-based thermal management materials, several limitations persist. First, thermochromic microcapsules primarily adjust within the solar spectral range, restricting their broader applicability. Second, the fabrication process is not sufficiently environmentally friendly, raising concerns about pollution. Lastly, thermochromic microcapsules are composed of complex organic materials with flammable properties, which poses safety challenges, particularly in the building sector [244].

##### Thermochromic Salinization Polymer

Although various thermochromic materials have been developed, each with distinct advantages, they often present limitations. For instance, inorganic thermochromic materials, such as VO_2_, have high switching temperatures, coloration issues, and high production costs. Organic thermochromic materials, such as thermochromic hydrogels, suffer from poor cycling stability and shorter lifespans, limiting their practical applications. Additionally, existing thermochromic materials face challenges such as difficult application processes, degradation over time, and restricted durability. Recently, a flexible thermochromic system comprising polyethylene oxide (PEO), polydimethylsiloxane (PDMS), and lithium perchlorate (LiClO_4_) has been reported (Fig. 8c). It combines properties of both organic and inorganic thermochromic materials, achieving a strong thermochromic effect along with excellent stability, processability, and biocompatibility, making it highly promising for extended applications [189].

#### Temperature Response

Certain materials exhibit changes in physical and chemical properties with variations in temperature, and this characteristic has led to their application in thermal management. Many dynamic radiative thermal management materials with temperature-responsive properties have been developed based on this principle [245, 246]. Compared with thermochromic materials, temperature-responsive dynamic radiative thermal management materials share some similarities but also have distinct differences. Both types of materials switch thermal management modes in response to temperature changes; however, the mechanisms differ. Thermochromic materials switch modes through temperature-induced color changes, while temperature-responsive materials undergo changes in their intrinsic physical and chemical properties. Temperature-responsive materials can be classified based on these responsive properties into categories such as electromagnetic, mechanical deformation, refractive index, and thermal resistance [247, 248]. For example, by integrating a radiative cooling coating with a temperature-responsive component, such as a Nitinol spring, the thermal resistance changes with temperature, allowing the thermal management mode to be switched by modulating heat emission [234] (Fig. 8d). In addition, phase change materials, as a special type of temperature-responsive material, also have great potential for applications in maintaining temperature comfort and saving energy [249, 250]. This is due to the fact that phase change materials themselves undergo a phase transition in response to changes in external temperature, a process that requires the absorption or release of latent heat [251, 252]. Therefore, combining them with thermal management materials can greatly facilitate the application of different thermal management modes [253].

#### Photochromic

Some materials also exhibit changes in optical properties when exposed to light, enabling thermal management mode switching [254, 255]. Based on their mechanism of action, these materials can be divided into two categories: physical and chemical. Physical action involves a physical change, such as in shape memory materials that alter optical properties under light exposure [256]. In contrast, chemical action is seen in photochromic materials where light-induced chemical structural changes lead to color variations. Photochromic materials can be further classified as inorganic or organic, with inorganic photochromic materials being widely studied due to their superior durability [257, 258]. For example, a flexible photochromic film with high brightness, transmittance, and low haze was developed by in situ growth of highly dispersed, small-sized Cu-doped WO_3_ nanoparticles within a poly(methyl methacrylate) matrix (Fig. 8e). This film’s light transmittance adjusts with solar intensity without requiring additional energy input, showing potential for large-scale applications [235].

#### Humidity Response

Previous studies have revealed materials responsive to artificial humidity stimulation; however, this type of control requires human intervention, which increases both application costs and the complexity of large-scale implementation. In light of the rapid advancements in wearable technologies and the variable sweat emissions of the human body in different environments [259], researchers have developed numerous smart textiles with adaptive hygrothermal management capabilities. These textiles can automatically adjust their heat transfer properties based on moisture levels to help maintain human body comfort [260–262]. One example is a humidity-triggered infrared-adaptive textile created by coating carbon nanotubes onto triacetate-cellulose bimorphic fibers and has been demonstrated to achieve over 35% modulation of infrared radiation in response to changes in relative humidity [236] (Fig. 8f). To further enhance suitability for large-scale production, a humidity-responsive, shutter-like textile with dynamic radiative thermal management properties was designed. This textile incorporates moisture-sensitive, high-twist single-helix yarns woven into spring-like chiral loops (Fig. 8g). When the fabric is exposed to humidity, the internal woven voids expand, accelerating the release of water vapor and heat. Conversely, in cold or dry environments, the woven rings contract, closing the gaps and reducing heat emission. Notably, this type of blinds-style fabric is manufactured using industrial textile technology, fully compatible with traditional textile processing and suitable for mass production [237].

## Practical Applications

### Personal Thermal Management

**Fig. 9:**
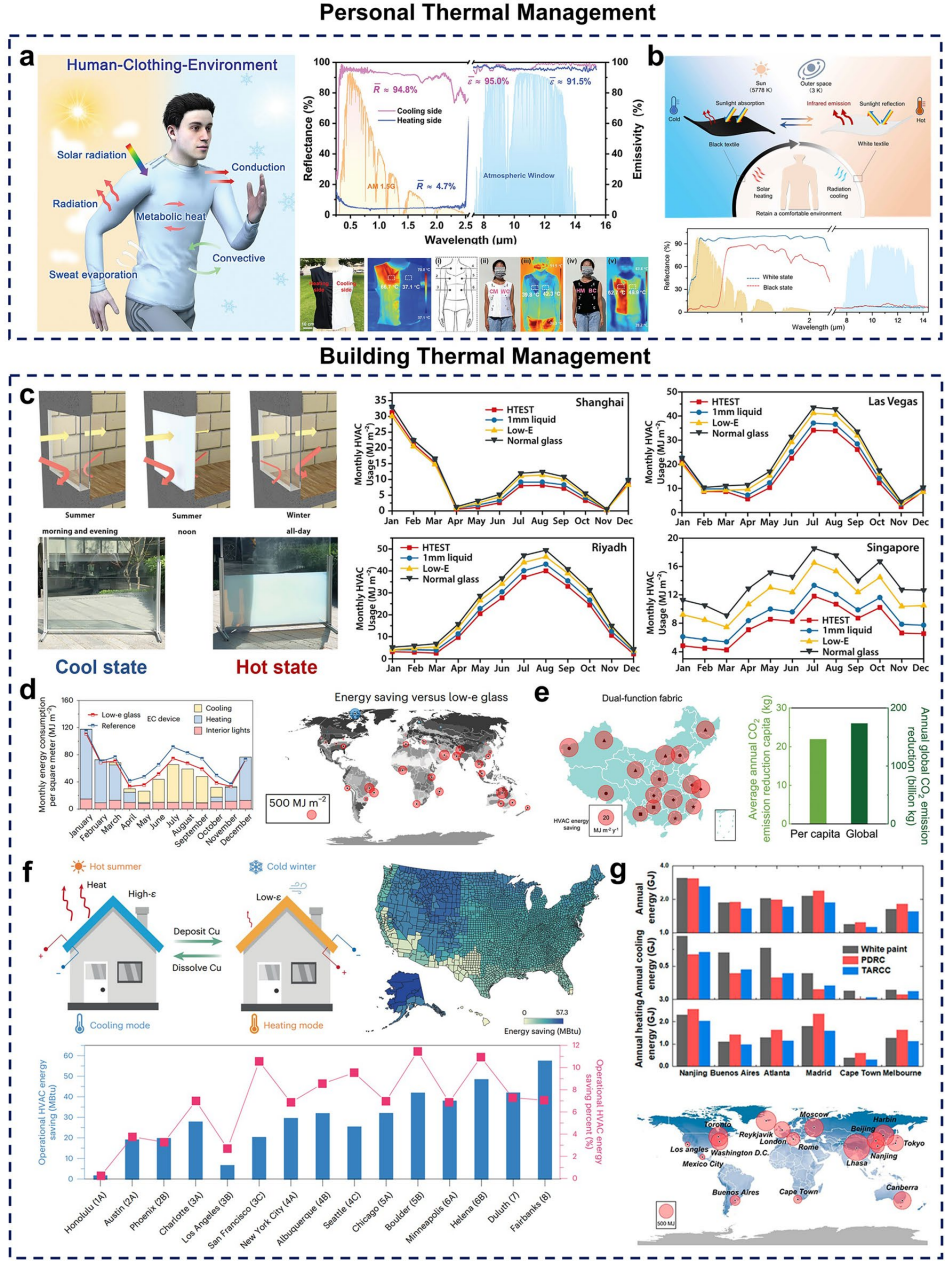
**a**, **b** Dynamic radiative thermal management applications in personal thermal management. **a** Active thermal management fabrics with switchable properties. [273] Copyright 2024, Wiley–VCH. **b** Passive adaptive thermal management fabrics. [274] Copyright 2024, AAAS. **c-g** Dynamic thermal management applications in energy-efficient buildings. **c** Thermochromic hydrogel-based smart window and energy-saving effects. [275] Copyright 2020, Elsevier. **d** Electrochromic smart window and energy-saving effects. [136] Copyright 2024, Springer Nature. **e** Sandwich fabrics for dynamic radiant thermal management of roofs. [276] Copyright 2023, Wiley–VCH. **f** Electrochromic device for building fences and energy-saving outcomes. [141] Copyright 2024, Springer Nature. **g** Adaptive coatings for building exteriors and energy-saving effects. [277] Copyright 2023, American Chemical Society

Maintaining thermal comfort is essential for the human body to perform normal physiological functions and daily activities, making body temperature regulation critical [263, 264]. As textiles serve as the interface between human skin and the environment, they play a key role in maintaining and providing comfort [265]. However, with the effects of global warming and an increase in extreme environments, traditional textiles are often inadequate for adapting to complex environmental conditions, particularly in very cold or hot climates. This limitation not only affects wearer comfort but also necessitates the use of conventional thermal management devices for temperature regulation, which can further increase energy consumption [266, 267]. To address this challenge, a variety of smart textiles have been developed, some of which offer basic dynamic radiative thermal management, while others are capable of autonomously regulating heat and moisture dissipation in response to environmental changes [268]. Based on their energy requirements, smart thermoregulation textiles are classified into two main categories: active and passive, with active textiles typically requiring external energy input, while passive textiles adapt spontaneously to environmental changes without additional energy [269–272]. For instance, an asymmetric leather nanotextile based on polyurethane (PU) material has been developed using electrospinning technology. It enables dual-mode switching between cooling and heating through simple physical flipping (Fig. 9a). Wear tests have shown that it can create a comfortable microenvironment for the body under varying weather conditions, with good breathability, softness, stretchability, and moisture-wicking capabilities [273]. However, this material requires manual flipping, which can be inconvenient compared to dynamic fabrics that respond automatically to environmental stimuli, enabling spontaneous thermal management mode switching without external energy. To advance autonomous thermoregulation, Zhu et al. incorporated thermochromic microcapsules into textile fibers, creating a bimodal photonic textile capable of low-temperature SH and high-temperature radiative cooling under sunlight (Fig. 9b). This textile offers visible light modulation of approximately 80%, extending the thermal comfort range for the wearer by up to 8.5 °C, and can be easier for production and scalability [274].

For smart thermoregulation textiles to reach commercial viability, several challenges must be addressed. First, production costs need to be reduced without compromising performance. Second, it is crucial to understand and meet consumer expectations. Finally, ongoing testing and optimization of textile performance are essential to ensure that large-scale production aligns with sustainable and economically viable practices.

### Building Thermal Management

#### Smart Windows

With rapid global population growth and urbanization, fossil fuel consumption continues to increase, heightening the urgency for energy conservation and environmental protection [278]. According to some existing statistics, building energy consumption accounts for approximately 30% to 40% of total global energy use and contributes to about 10% of total annual carbon emissions, with 60% of this energy dedicated to traditional heating and cooling, a trend that continues to increase annually [279, 280]. Additionally, climate change and population growth are projected to drive an 80% increase in building temperature-controlled energy consumption from 2010 to 2050, making the development of sustainable radiative thermal management technologies to reduce fossil fuel use and carbon emissions an urgent priority [281]. As a key component of buildings, windows play an essential role in lighting and regulating heat exchange. However, they are also one of the least energy-efficient parts of buildings, accounting for around 4% of total heating and cooling energy consumption. Therefore, improving the energy efficiency of windows is vital to achieving overall building energy efficiency [282]. To address this need, researchers have developed various smart windows that can switch between different optical states, adjusting transparency in response to external stimuli and enabling dynamic radiative thermal management [283]. For example, by incorporating thermochromic hydrogel-derived liquid into glass, simulations demonstrated a 44.6% reduction in HVAC energy consumption compared to standard glass, with promising energy-saving potential across various urban environments [275] (Fig. 9c). Although this approach is straightforward, homogeneous, and highly scalable, issues such as potential water leakage and drying within the smart window require further attention. To expand the spectral modulation range, an electrochromic smart window with adaptive thermal radiation properties across the visible, near-infrared, and MIR regions was developed [136]. Compared to standard commercial glass, this device achieved energy savings of up to 596.7 MJ m⁻^2^ across a variety of climatic conditions, highlighting its potential for substantial energy efficiency improvements (Fig. 9d).

#### Roofs

Roofs are an extremely important part of a building’s structure, playing an important role in protecting it from environmental elements such as wind and rain. However, traditional roofing materials are typically static, meaning their thermal management properties are fixed and unable to adapt to changing seasons and weather conditions [284]. Studies have shown that in summer, up to 40%–60% of heat enters a building through the roof, easily raising indoor temperatures and increasing the energy demand for air conditioning [285]. Even with modern insulation materials, the persistently high temperatures on roof surfaces during hot seasons can reduce the durability of roofing materials. In winter, traditional roofing may not absorb sufficient sunlight and can also lose heat through radiation, resulting in lower indoor temperatures. Thus, dynamic radiative thermal management techniques for building roofs are essential to improve energy efficiency and comfort. To address this, researchers have developed a range of innovative materials and functional coatings to replace conventional roofing, allowing for seasonal adaptability to achieve cooling in summer and warmth in winter [286]. Assembly of vertical graphene, graphene glass fiber fabric, and polyacrylonitrile nanofibers led to the development of a thermal management fabric with a sandwich structure [276]. Using it on a building roof, related simulation results showed that this fabric could reduce global CO_2_ emissions by about 173.7 MT per year compared to a similar product with a single thermal management property (Fig. 9e).

#### Fences

Building fences contribute significantly to safety, environmental protection, and aesthetic enhancement. However, traditional fencing materials tend to have high solar absorption, which increases cooling energy demands in hot seasons [287]. The heat exchange facilitated by building fence structures represents a considerable portion of overall building energy consumption [288]. Therefore, developing fencing materials with thermal management capabilities is essential as an alternative to conventional fences, aiming to reduce energy consumption and mitigate the urban heat island effect [289, 290]. Sui et al. developed an aqueous electrochromic structure for building fences that demonstrates excellent tunability in thermal emissivity [141]. Simulation results indicate that this fencing structure could achieve an average annual HVAC energy savings of approximately 8.4% across climate zones 5–8 in the USA (Fig. 9f). However, the high manufacturing cost currently limits its large-scale application, and further efforts are needed to reduce raw material and production costs to enable mass production.

#### Coatings

Architectural coatings are materials applied to building surfaces, forming a strong, cohesive protective layer that bonds well with the substrate. These coatings serve not only decorative functions, enhancing the building’s aesthetic appeal, but also protecting it from environmental impacts and damage [291, 292]. However, with recent technological advances and increasingly extreme climate variations, traditional coatings are becoming inadequate to meet people’s diverse needs. Although various thermal management coatings have been developed, most focus solely on radiant cooling and are limited in their ability to provide both warmth in winter and cooling in summer [293–295]. This limitation underscores the need for intelligent thermal management coatings capable of adapting to temperature changes. Inspired by the adaptive color change of chameleons, a temperature-responsive coating was developed by incorporating thermochromic microcapsules. This coating is low cost, easy to produce, and straightforward to apply (Fig. 9g). Simulation results indicate that it can save up to 20% of annual energy and extend comfortable working hours in mid-latitude regions compared to PDRC coatings [277].

### Vehicle Thermal Management

**Fig. 10:**
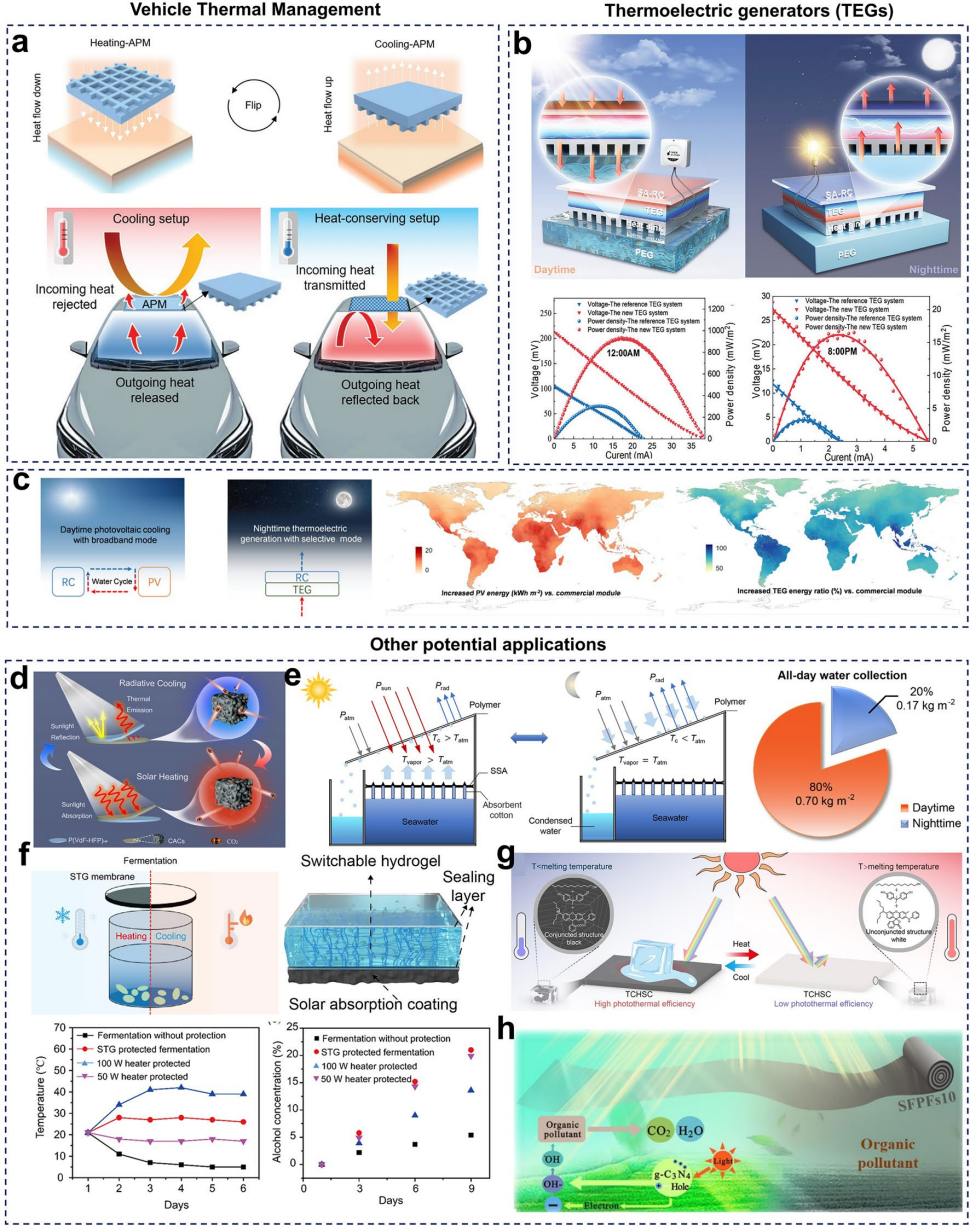
**a** Dynamic radiative thermal management applied to vehicle thermal management. [99] Copyright 2022, Wiley–VCH. **b, c** Dynamic radiative thermal management in thermoelectric generators (TEG). **b** Phase change material-based TEG for all-day sustainable power supply. [297] Copyright 2024, Wiley–VCH. **c** Simulation of power generation efficiency of an infrared-adaptive radiative cooling device for temperature difference power generation. [298] Copyright 2024, RSC. **d-h** Potential applications of dynamic radiative thermal management. **d** CO_2_ variable temperature adsorption. [299] Copyright 2022, Elsevier. **e** All-weather freshwater harvesting. [300] Copyright 2022, American Chemical Society. **f** Temperature control in alcohol fermentation. [301] Copyright 2023, Elsevier. **g** Smart de-icing. [188] Copyright 2024, Elsevier. **h** Thermo-photogate reaction system for photocatalytic applications. [302] Copyright 2022, Elsevier

Vehicles have become integral to our daily lives, but extreme temperatures during hot summers or cold winters can cause significant fluctuations in the interior temperature, impacting driver comfort and, in severe cases, posing safety risks [35]. To maintain a comfortable environment, drivers often rely on temperature control systems, such as on-board air conditioning, which increases energy consumption. With the rapid growth and adoption of new energy vehicles, effective vehicle thermal management has become an urgent priority [296]. In response, Ly et al. developed a dual-mode APM designed for all-season thermal management in outdoor vehicles (Fig. 10a), which allows vehicles to maintain a cool interior during hot seasons by transmitting thermal radiation broadband and reflecting ambient heat. During colder periods, the thermal pathway reverses, helping to retain warmth inside the vehicle [99].

### Thermoelectric Generators (TEGs)

TEG is an energy conversion device that harnesses the temperature difference between hot and cold ends to directly generate electricity, based on the Seebeck effect [303–305]. With recent advancements in thermal management, the concept of using renewable energy sources for power generation has attracted significant research interest. SH technology, when coupled with TEGs, generates a temperature differential for power generation during daylight hours but is unable to sustain power production at night. PRC techniques can facilitate all-day power generation, though the output voltage is often very low [306, 307]. Thus, developing an efficient, sustainable, and continuous power generation system that does not require energy storage remains a challenge. To address this, researchers have introduced spectrally adaptive phase change materials to both sides of the TEG device. The phase change materials on the top layer enable automatic switching of thermal management modes, while those on the bottom expand the spatial temperature difference, enhancing thermoelectric power density and enabling continuous, all-day power generation (Fig. 10b). The experimental results demonstrated that this innovative TEG device achieved a power density increase of 123.1% during the day and 249.1% at night compared to conventional designs [297]. Although most of the research reports have realized all-weather power generation, it is still unknown how efficient the power generation is in large-scale applications [308, 309]. Recently, Tang et al. has successfully prepared an infrared-adaptive radiative cooler using thermo-responsive hydrogel as raw material [298]. It can increase the efficiency of PV power generation by 12% during the daytime, and promote the efficiency of TEG power generation by 80% at night. Simulation results further show that the PV-TEG hybrid system with this radiative cooler can increase the daytime power generation by 20 kWh m^−2^ and double the thermoelectric power at night (Fig. 10c).

### Other Potential Applications

In addition to the above applications discussed, dynamic radiative thermal management holds promise for a variety of other applications, which are introduced and analyzed here to provide insights for future advancements in this technology.

Variable temperature adsorption is an effective technology for CO_2_ capture, though the process is typically energy intensive. By integrating PRC and SH techniques, the energy consumption of adsorption/desorption systems can be significantly reduced, achieving performance comparable to conventional adsorption systems [299] (Fig. 10d). Solar-driven interfacial evaporation for water harvesting achieves high evaporation rates and is environmentally sustainable; however, conventional interfacial evaporation suffers from significant thermal radiation and heat transfer losses from the bulk water to the environment. Additionally, the absence of sunlight limits the ability to collect freshwater at night. In contrast, integrating selective SH and PRC enables freshwater harvesting under all-weather conditions, offering promising applications in desalination and freshwater collection in tropical desert regions [300] (Fig. 10e). In addition, dynamic radiative thermal management materials have demonstrated the capability to regulate temperatures for alcoholic fermentation, enable intelligent de-icing, and function as thermo-photogate systems in photocatalytic reactions [188, 301, 302] (Fig. 10f-h). These potential applications not only broaden the scope of dynamic radiative thermal management technology but also accelerate its progress toward large-scale implementation.

## Conclusion and Challenges

**Fig. 11:**
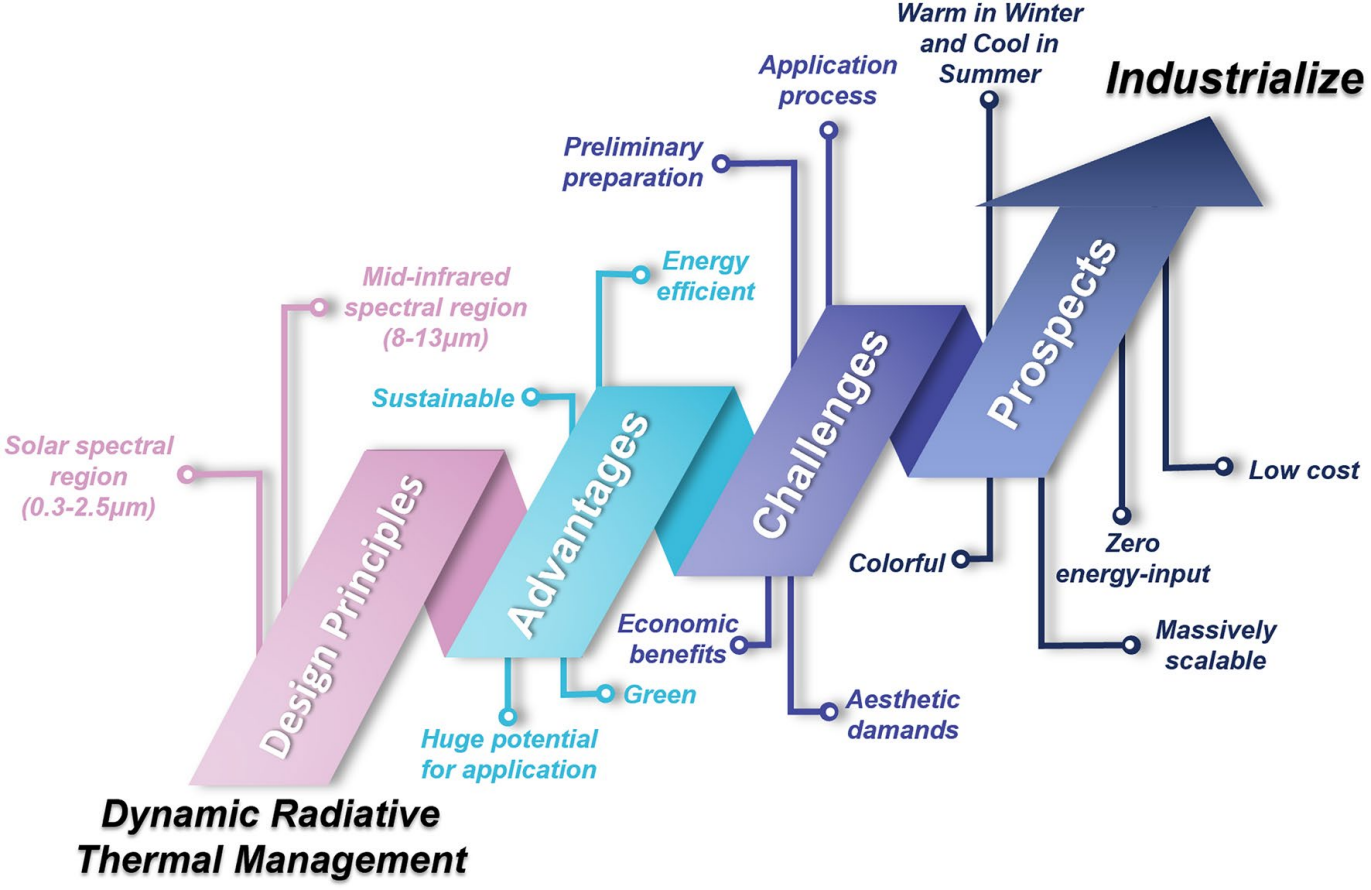
Practical challenges and prospects for large-scale applications of dynamic radiative thermal management

A single thermal management approach is increasingly inadequate for addressing the complex and evolving external environment as well as the diverse needs of daily life. To adapt to these dynamic conditions, the concept of dynamic radiative thermal management has emerged as an innovative solution. This paper reviews recent advancements in dynamic radiative thermal management, providing a comprehensive overview of its basic principles, methodologies, related applications, and existing challenges. Dynamic radiative thermal management materials are categorized by their mode switching mechanisms in practical applications into two types: active and passive. Active systems require external energy input or an intermediary and include approaches such as physico-mechanical action, electrochromic response, and chemical stimulation. In contrast, passive systems can respond autonomously to changes in temperature, humidity, or light, switching thermal management modes without additional energy input. Currently, dynamic radiative thermal management applications span areas such as personal thermal management, energy-efficient buildings, vehicle thermal management, and thermoelectric power generation, with a range of potential applications awaiting further exploration and expansion. Despite substantial progress in this field, several challenges remain for practical scaleable implementation (Fig. 11).

Firstly, regarding the prerequisite preparation for practical applications, current demonstrations of dynamic radiative thermal management applications largely rely on conceptual simulation experiments, which are highly idealized to some extent. In real-world conditions, environmental factors are complex and variable, and there remains a considerable gap in research on large-scale practical applications. Bridging the gap between laboratory proof-of-concept studies and practical applications is therefore essential. Furthermore, existing dynamic radiative thermal management materials do not yet reach the ideal spectral modulation capabilities and further refinement is still needed. Additionally, testing methods and results across current studies are varied, with no standardized methods or reference standards in place, making evaluations of actual thermal management performance less accurate. Secondly, from a practical application standpoint, dynamic radiative thermal management technology is highly dependent on natural conditions. Small changes in solar radiation or atmospheric conditions can affect the stability of thermal management performance, yet strategies to address this limitation have not been fully explored. Furthermore, in urban environments, dynamic radiative thermal management materials may be blocked by high-rise buildings, reflecting energy in ways that could inadvertently worsen the urban heat island effect [310]. Another issue that cannot be overlooked is that real-world applications will inevitably encounter rain, snow, hail, and other challenging weather conditions, making it necessary to improve the corrosion resistance, aging resistance, and stability of these materials. For personal thermal management applications, factors such as wearing comfort and environmental friendliness must also be considered. From an economic perspective, most manufacturing processes for dynamic radiative thermal management materials involve complex and costly methods, which are not suitable for mass production. Reducing production costs and simplifying manufacturing processes are thus necessary prerequisites for large-scale applications. Finally, from an aesthetic perspective, current dynamic radiative thermal management materials lack sufficient color variety. Enhancing the aesthetics of these materials to meet users’ aesthetic and cultural expectations, without sacrificing optical performance, remains an ongoing challenge.

In summary, although dynamic radiative thermal management technology holds significant application prospects and potential, it still has a long way to go before it can be implemented in actual production, and achieving this will require the joint efforts of researchers in related fields.
